# Adaptive power-saving mode control in NB-IoT networks using soft actor-critic reinforcement learning for optimal power management

**DOI:** 10.1038/s41598-025-18214-4

**Published:** 2025-10-03

**Authors:** S. Anbazhagan, R. K. Mugelan

**Affiliations:** Department of Communication Engineering, School of Electronics Engineering, Vellore Institute of Technology, Katpadi, Vellore, Tamilnadu 632014 India

**Keywords:** Narrowband internet of things (NB-IoT), Power efficiency, Power-saving modes, Reinforcement learning, Soft-actor critic (SAC), Engineering, Electrical and electronic engineering

## Abstract

In the evolving landscape of the Internet of Things (IoT), optimizing power efficiency in Narrowband IoT (NB-IoT) networks is crucial for extending device lifetimes while maintaining performance. This research leverages the Soft Actor-Critic (SAC) reinforcement learning algorithm to intelligently manage power-saving modes in NB-IoT devices. The study compares SAC with Proximal Policy Optimization, and Deep Q-Network. The methodology involves simulating an NB-IoT environment and evaluating performance using metrics such as total reward, overall energy efficiency, power consumption, mode count and duration, and duty cycle percentage. The SAC-based approach demonstrated significant improvements in power efficiency, achieving balanced enhancements in power conservation and network performance. These findings suggest that reinforcement learning techniques like SAC can play a pivotal role in advancing the efficiency and sustainability of NB-IoT networks, leading to prolonged device operation, reduced costs, and enhanced overall performance, thus paving the way for more resilient and scalable IoT deployments.

## Introdution

The rapid expansion of the Internet of Things (IoT) has ushered in a multitude of applications, from smart homes and industrial automation to healthcare and environmental monitoring. These diverse applications generally require low data rates, prolonged battery life, and low power consumption. Simultaneously, they necessitate extensive coverage and the capacity to support a vast number of devices^1^. As the number of connected devices continues to surge, there is an urgent need for communication technologies that are both robust and scalable to handle this growth. Low Power Wide Area (LPWA) networks have emerged as a promising solution to meet these requirements. LPWA networks are engineered to deliver widespread connectivity with minimal data rates and power consumption. They can operate on either licensed or unlicensed spectrum and are powered by both cellular and non-cellular technologies^2^. Among the various LPWA technologies, Narrowband Internet of Things (NB-IoT) stands out as a leading solution. NB-IoT, standardized by the Third Generation Partnership Project (3GPP) in Release 13, operates in a narrow bandwidth of 180 kHz. It can be integrated into existing LTE networks, deployed in a re-farmed GSM spectrum, or established as a standalone network^3^. This versatility makes NB-IoT an attractive option for a broad array of IoT applications that demand wide coverage and low data rates. NB-IoT is particularly suitable for applications such as e-health, smart agriculture, smart cities, smart parking, logistics, and waste management. These applications typically involve the transmission of small, infrequent, and delay-tolerant data, making NB-IoT an optimal choice. The technology facilitates quick and efficient deployment, as it can coexist with existing LTE infrastructure^4^. Additionally, NB-IoT offers advanced features like mass communication, restricted mobility support, and accurate positioning, broadening its range of applications. By operating in licensed frequency bands, NB-IoT minimizes interference, ensuring reliable communication for critical use cases^5^.

### Primary goals of NB-IoT

#### Extended coverage

NB-IoT is engineered to deliver enhanced coverage, especially in challenging environments such as deep indoors or underground locations. This capability is crucial for applications like smart metering, environmental monitoring, and infrastructure management, where devices may be situated in areas with poor signal penetration. With support for a Maximum Coupling Loss (MCL) of 164 dB, NB-IoT offers over 20 dB deeper coverage compared to legacy LTE, ensuring connectivity in remote or densely built environments. This extended reach significantly expands the potential use cases of IoT technologies, enabling reliable operation in scenarios that were previously difficult to service^6^.

#### Low power consumption

A hallmark feature of NB-IoT is its ability to support devices with extended battery life, potentially lasting up to 10 years on a single battery. This is achieved through various power-saving techniques and optimized communication protocols that minimize energy usage. NB-IoT supports ultra-low complexity by simplifying the device structure and reducing the network protocol volume, resulting in lower energy consumption and decreased device cost. Low power consumption is particularly important for devices deployed in locations where frequent battery replacement is impractical or costly, such as remote sensors or asset tracking devices^7^.

#### High device density

NB-IoT can support a high density of devices per cell, often accommodating thousands of devices within a single network cell. It supports more than 52,500 connections per cell, making it essential for urban and industrial environments where a large number of IoT devices need to communicate simultaneously. This high capacity ensures that NB-IoT can handle a significant number of devices without substantial interference or congestion, making it suitable for applications in smart cities, industrial automation, and agricultural monitoring^8^.

#### Cost efficiency

The cost of NB-IoT modules is relatively low compared to other cellular IoT technologies, and the ability to leverage existing cellular infrastructure reduces deployment costs. This cost efficiency makes NB-IoT an attractive option for widespread IoT deployments across various sectors. Lower costs not only facilitate the adoption of IoT technologies by businesses and governments but also enable innovative applications in areas such as healthcare, transportation, and public safety. By supporting ultra-low complexity and simplifying the device structure, NB-IoT further reduces the cost of devices, making it accessible for a broad range of applications^9^.

#### Interoperability and coexistence

NB-IoT is designed to coexist seamlessly with existing LTE networks, allowing operators to deploy NB-IoT alongside their current cellular infrastructure without the need for significant modifications. This coexistence ensures that NB-IoT can be implemented rapidly and cost-effectively. Furthermore, NB-IoT supports interoperability with other IoT technologies, facilitating smooth integration into a wide range of applications and systems. This interoperability is crucial for developing a unified IoT ecosystem where various devices and platforms can communicate and operate together efficiently.

These features collectively make NB-IoT a robust, efficient, and cost-effective solution for a wide range of IoT applications, addressing the critical needs of connectivity, power efficiency, and scalability^10^.

### Importance of NB-IoT in the IoT ecosystem

NB-IoT is poised to play a transformative role in the IoT ecosystem due to its unique combination of features. It addresses several critical requirements for IoT deployments, including extensive coverage, long battery life, high device density, and cost efficiency. These attributes make NB-IoT particularly suitable for a range of applications, from smart cities and agriculture to industrial automation and environmental monitoring^11^.

#### Smart cities

In smart city initiatives, NB-IoT can be used for a multitude of applications, including smart parking, waste management, and street lighting. Its ability to provide reliable connectivity in dense urban environments and its low power requirements make it an ideal choice for city-wide sensor networks. For instance, NB-IoT can facilitate efficient traffic management by monitoring and controlling parking spaces, reducing congestion, and improving urban mobility. Additionally, smart street lighting systems can leverage NB-IoT to reduce energy consumption by adjusting lighting based on real-time data, enhancing both safety and sustainability^12^.

#### Industrial IoT

In industrial settings, NB-IoT enables applications such as asset tracking, predictive maintenance, and remote monitoring of machinery. Its robust connectivity and long battery life are essential for monitoring equipment in large factories or remote sites. By providing real-time data on machine performance and potential faults, NB-IoT supports predictive maintenance strategies, reducing downtime and maintenance costs. Furthermore, NB-IoT’s ability to support a high density of devices per cell is crucial for managing extensive industrial operations, ensuring seamless communication across various equipment and systems^13^.

#### Agriculture

NB-IoT can enhance agricultural productivity through applications like soil moisture monitoring, livestock tracking, and precision farming. Its extensive coverage ensures that sensors can communicate effectively even in vast, rural areas. Farmers can utilize NB-IoT to monitor soil conditions in real-time, optimizing irrigation and fertilization practices to improve crop yields and conserve resources. Livestock tracking systems enabled by NB-IoT provide valuable insights into animal health and behavior, facilitating better management and reducing losses. Precision farming techniques, supported by NB-IoT, allow for targeted interventions, increasing efficiency and sustainability in agriculture^14^.

#### Healthcare

In the healthcare sector, NB-IoT supports applications like remote patient monitoring, wearable health devices, and emergency response systems. Its low power consumption extends the operational life of health monitoring devices, reducing the need for frequent recharging or battery replacement. This is particularly beneficial for elderly or chronically ill patients who require continuous monitoring. NB-IoT enables the transmission of critical health data to medical professionals in real-time, enhancing patient care and enabling timely interventions. Additionally, wearable health devices powered by NB-IoT can track vital signs and activity levels, promoting proactive health management and early detection of potential issues^15^.

#### Environmental monitoring

NB-IoT is well-suited for environmental monitoring applications, including air quality monitoring, water quality assessment, and disaster detection. Its ability to provide reliable connectivity in remote and challenging environments makes it ideal for deploying sensors in forests, rivers, and coastal areas. Environmental monitoring systems utilizing NB-IoT can deliver real-time data on pollution levels, helping authorities take timely action to protect public health and the environment. In disaster detection and management, NB-IoT can play a critical role by providing early warnings for events such as floods, landslides, and wildfires, enabling swift responses to mitigate damage and ensure safety^16^.

#### Logistics and supply chain management

In logistics and supply chain management, NB-IoT enhances the tracking and management of goods throughout their journey. Its long battery life and extensive coverage make it possible to monitor the condition and location of shipments in real-time, even in remote areas. NB-IoT enables better inventory management, reduces the risk of loss or theft, and ensures that goods are transported under optimal conditions. This is particularly important for perishable goods, pharmaceuticals, and high-value items, where maintaining the integrity and security of the shipment is critical^17^.

#### Smart homes and buildings

NB-IoT contributes to the development of smart homes and buildings by enabling various automation and monitoring applications. From smart thermostats and security systems to energy management and appliance control, NB-IoT provides the connectivity needed to create efficient, secure, and comfortable living environments. Smart home devices powered by NB-IoT can communicate seamlessly, allowing residents to control and monitor their homes remotely. In commercial buildings, NB-IoT supports energy management systems that optimize heating, cooling, and lighting, reducing operational costs and environmental impact^18^.

NB-IoT’s extensive coverage, low power consumption, high device density, cost efficiency, interoperability, and coexistence with existing cellular networks make it a cornerstone of the IoT ecosystem. Its versatility and robust performance across various applications from smart cities and industrial automation to agriculture, healthcare, environmental monitoring, logistics, and smart homes highlight its transformative potential. As the IoT landscape continues to evolve, NB-IoT will play an increasingly vital role in enabling innovative solutions and driving the adoption of IoT technologies worldwide^19^.

### Challenges in power management for NB-IoT devices

Despite its many advantages, NB-IoT faces significant challenges, particularly in the area of power management. IoT devices are often deployed in remote or hard-to-reach locations where frequent battery replacement is impractical. As such, extending the battery life of these devices is crucial. Traditional power management methods often fail to adapt to the dynamic nature of IoT environments, resulting in suboptimal power consumption. These methods do not account for the varying network conditions, data transmission requirements, and operational contexts that can significantly impact the energy efficiency of NB-IoT devices.

Power-saving modes in NB-IoT, such as extended discontinuous reception (eDRX) and power-saving mode (PSM), are designed to reduce energy consumption by allowing devices to enter low-power states when not actively transmitting or receiving data. However, the effectiveness of these modes depends on the ability to dynamically and intelligently manage their activation and deactivation based on real-time network conditions and device states. Achieving this dynamic management is a complex task that requires advanced optimization techniques^20^.

### Importance of optimizing power-saving modes

Optimizing power-saving modes in NB-IoT devices is essential for several reasons. First, it directly impacts the operational lifetime of IoT devices, reducing the need for frequent battery replacements and maintenance, which can be costly and logistically challenging. Second, improved power efficiency enhances the overall sustainability of IoT deployments, contributing to lower energy consumption and reduced environmental impact. Third, optimizing power-saving modes ensures that devices maintain an acceptable level of performance, balancing energy savings with the need for reliable and timely data transmission^21^.

In this context, leveraging advanced machine learning techniques, such as reinforcement learning, offers a promising approach to optimize power management in NB-IoT networks. By using algorithms like Soft Actor-Critic (SAC), it is possible to develop intelligent power management strategies that dynamically adjust power-saving modes in response to changing network conditions and device states. This research aims to explore the potential of SAC-based reinforcement learning to enhance power efficiency in NB-IoT networks, demonstrating significant improvements over traditional methods and other reinforcement learning algorithms such as Proximal Policy Optimization (PPO), and Deep Q-Networks (DQN).

### Problem statement

In NB-IoT networks, the efficient management of power consumption poses a critical challenge due to several inherent factors. Firstly, these networks often rely on battery-powered IoT devices, which inherently have limited energy reserves. Consequently, prolonging the operational lifespan of these devices while maintaining their functionality becomes imperative. Secondly, NB-IoT devices operate in diverse and dynamic environments, where network conditions can fluctuate significantly. These conditions include variations in signal strength, interference levels, and traffic load, all of which impact power consumption. As a result, devising strategies to adapt to these changing conditions while optimizing power usage presents a complex problem. Moreover, NB-IoT networks typically consist of heterogeneous devices with diverse characteristics and requirements. Some devices may prioritize low latency for real-time applications, while others may prioritize energy efficiency for prolonged battery life. Balancing the needs of these diverse devices to achieve optimal power efficiency without sacrificing individual performance further complicates the issue.

### Need for intelligent power-saving mode selection

The need for intelligent power-saving mode selection in NB-IoT networks arises from several key considerations. Firstly, maximizing device uptime is paramount to ensure continuous operation and minimize disruptions. By dynamically selecting appropriate power-saving modes based on real-time network conditions and device requirements, NB-IoT devices can remain operational for extended periods without manual intervention. This is particularly crucial in applications where devices are deployed in remote or inaccessible locations, where frequent battery replacements or recharging may not be feasible. Secondly, intelligent power-saving mode selection plays a vital role in optimizing energy consumption across the network. By leveraging adaptive algorithms and predictive analytics, NB-IoT devices can intelligently adjust their power-saving modes to match the current workload and environmental conditions. For example, during periods of low activity or low network demand, devices can switch to energy-saving modes to conserve power. Conversely, when network traffic increases or latency-sensitive tasks are required, devices can seamlessly transition to higher-power modes to meet performance requirements^22^.

Furthermore, intelligent power-saving mode selection enhances the overall reliability and resilience of NB-IoT networks. By strategically managing power consumption, devices can ensure consistent connectivity and data transmission even in challenging environments. For instance, devices can prioritize power-intensive operations during periods of optimal signal strength and conserve power during times of interference or congestion. This adaptive approach not only improves network reliability but also reduces the risk of service disruptions due to battery depletion or network congestion. In essence, intelligent power-saving mode selection is essential for optimizing the performance, efficiency, and reliability of NB-IoT networks in dynamic and heterogeneous environments. By leveraging advanced algorithms and adaptive strategies, these networks can achieve the delicate balance between power conservation and operational effectiveness, ultimately driving the widespread adoption and success of NB-IoT technologies^23^.

### Objectives

This research sets out to address the challenge of optimizing power efficiency in NB-IoT networks through intelligent power management. Utilizing advanced machine learning techniques, particularly reinforcement learning, such as Soft Actor-Critic (SAC), the aim is to develop adaptive strategies for selecting and adjusting power-saving modes in real-time. By dynamically responding to changing network conditions and device states, the overarching objective is to maximize energy conservation while ensuring optimal network performance. Moreover, the research seeks to conduct a comprehensive comparative analysis of various reinforcement learning algorithms in the context of power management in NB-IoT networks. Algorithms such as Proximal Policy Optimization (PPO), and Deep Q-Networks (DQN). will be evaluated to identify the most effective approach for achieving the desired objectives.

A key goal of the study is to demonstrate the superiority of Soft Actor-Critic (SAC) over traditional methods and other reinforcement learning algorithms. Through empirical evaluation and performance comparison, the aim is to showcase SAC’s capabilities in adapting to dynamic environments, handling continuous action spaces, and achieving higher levels of power efficiency and network reliability. Furthermore, the research aims to validate the effectiveness of the proposed SAC-based approach through simulation and experimentation. By implementing the developed algorithms in realistic NB-IoT network scenarios and conducting extensive testing, the objective is to provide empirical evidence of their efficacy in real-world deployment. Ultimately, the research aims to contribute to the advancement of intelligent power management techniques in NB-IoT networks. By offering novel insights, practical methodologies, and empirical evidence, the objective is to facilitate the adoption of SAC-based reinforcement learning and pave the way for more energy-efficient and reliable IoT deployments.

### Research findings

This study employs the Soft Actor-Critic (SAC) reinforcement learning algorithm to intelligently manage power-saving modes in NB-IoT devices. Comparative analysis is conducted with other algorithms including Proximal Policy Optimization (PPO), and Deep Q-Network (DQN). The methodology involves simulating an NB-IoT environment and evaluating performance using metrics such as total reward, overall energy efficiency, power consumption, mode count and duration, and duty cycle percentage.

The SAC-based approach demonstrated significant improvements in power efficiency, achieving balanced enhancements in power conservation and network performance. These findings suggest that reinforcement learning techniques like SAC can play a pivotal role in advancing the efficiency and sustainability of NB-IoT networks, leading to prolonged device operation, reduced costs, and enhanced overall performance, thus paving the way for more resilient and scalable IoT deployments.

## NB-IOT: key technology and its significance

**Fig. 1:**
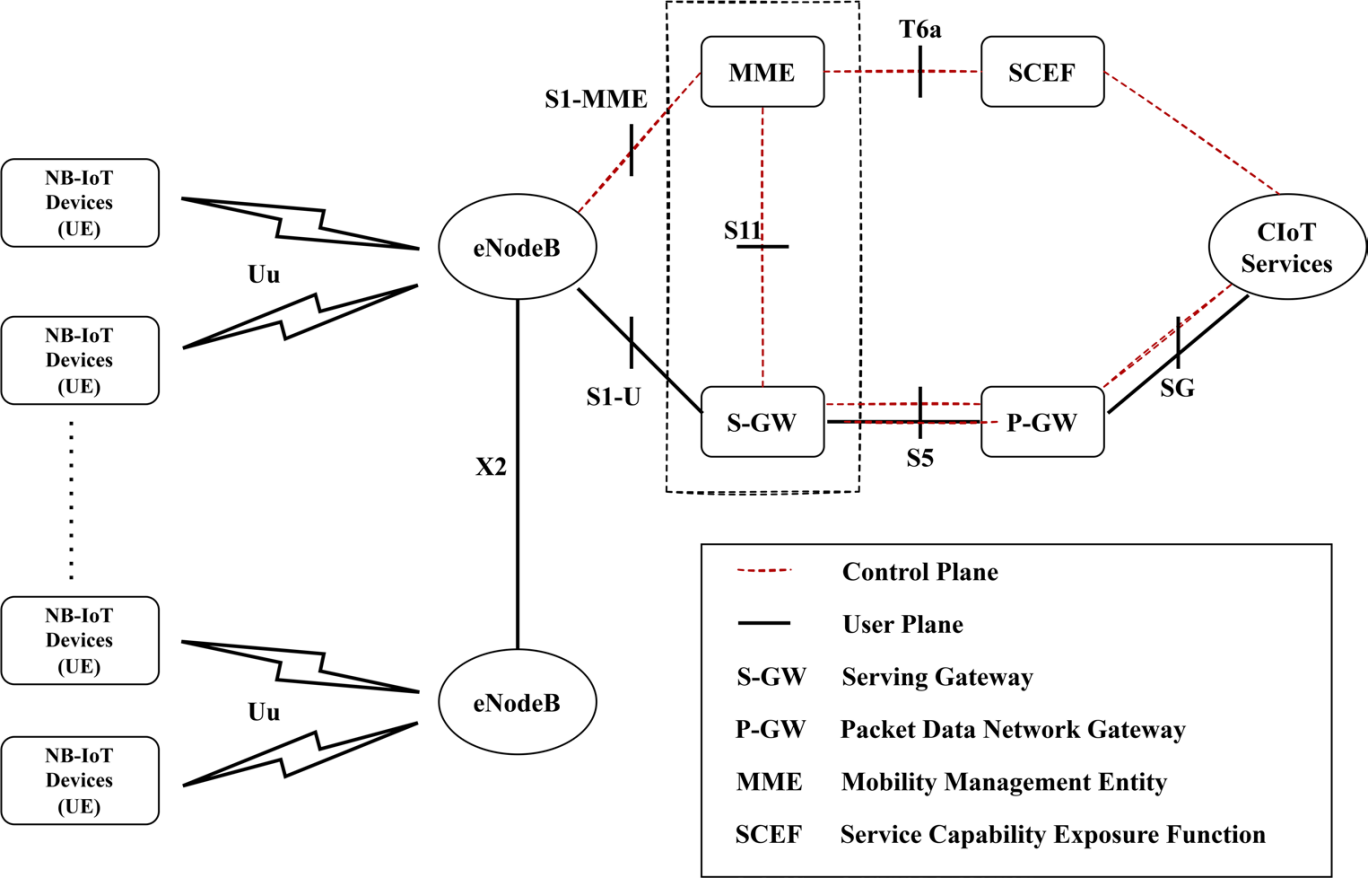
Architecture of the NB-IoT system showing core components, interfaces, and deployment modes for low-power IoT.

As illustrated in Fig. 1 architecture of Narrowband IoT (NB-IoT) is designed for the efficient connection of low-power devices across wide areas, utilizing existing cellular networks. At its core, NB-IoT includes User Equipment (UE), which are devices optimized for intermittent, low-power data transmission. The Radio Access Network (RAN) manages the communication between UE and the core network, supporting three deployment modes: Standalone (using dedicated spectrum), Guardband (using unused resource blocks within an LTE carriers guard band), and In-band (sharing spectrum with LTE carriers). The core network features several crucial components: the Mobility Management Entity (MME) handles signaling and control, the Serving Gateway (S-GW) routes and forwards user data packets, and the Packet Data Network Gateway (P-GW) connects the NB-IoT network to external IP networks. The Home Subscriber Server (HSS) stores user profiles and manages authentication and access, while the Service Capability Exposure Function (SCEF) optimizes IoT data transmissions and exposes services securely. Additionally, the IP Short Message Gateway (IP-SM-GW) supports SMS over IP networks^24^.

NB-IoT can optimize data transmission through Control Plane CIoT EPS Optimization, which reduces overhead for small, infrequent data transfers, or User Plane CIoT EPS Optimization for higher throughput requirements. Its roaming architecture supports seamless operation across different network operators via direct, indirect, and hybrid models that involve the SCEF for secure communication. The attached procedures offer options to establish or forego a Packet Data Network (PDN) connection during the attach process, catering to various device connectivity needs. Key features like Power Saving Mode (PSM), Extended Discontinuous Reception (eDRX), and Non-IP Data Delivery (NIDD) are integral to NB-IoT. PSM and eDRX enable devices to enter deep sleep states to save power, waking periodically to check for data. NIDD facilitates efficient data transmission without IP encapsulation, reducing overhead^25^.

The architecture also involves several key interfaces that ensure seamless communication and data transfer across the network. These include Um / Uu / LTE-Uu interfaces for connecting UE to the RAN, S1-MME, and S1-U interfaces for linking the RAN to the MME and S-GW respectively, and the S6a interface for connecting the MME to the HSS. The S11 interface connects the MME to the S-GW, while S5/S8 interfaces link the S-GW to the P-GW, with S5 used within a single network and S8 for roaming scenarios. The SGi interface connects the P-GW to external packet data networks, enabling broader communication. Interfaces like T6a/T6b/T6ai/T6bi support interactions between the SCEF and the P-GW or S-GW, enhancing non-IP data delivery and control plane optimization. The T7 interface facilitates communication between the home network’s SCEF and the visited network’s Interworking Function (IWF)-SCEF during roaming. SGd and Gd interfaces handle SMS delivery over the IP-SM-GW, supporting messaging services, while APIs enable standardized interactions between the SCEF and external application servers. These components and interfaces collectively ensure that NB-IoT is scalable and efficient, enabling massive device connectivity with minimal power consumption and operational costs^26^.

NB-IoT standard is tailored for the Internet of Things (IoT), offering robust technical specifications and features. It supports three deployment modes in-band, guard-band, and standalone allowing flexibility in utilizing existing LTE carriers and dedicated spectrum. NB-IoT operates across various global frequency bands, such as Bands 3, 8, and 20 in Europe and Bands 2, 4, 5, and others in North America and the Asia Pacific. With a narrow bandwidth of 180 kHz, NB-IoT excels in providing extensive indoor and long-range coverage, potentially up to 120 km, by employing enhanced NPRACH formats and small-cell support. The technology emphasizes low power consumption, featuring Power Saving Mode (PSM) and extended Discontinuous Reception (eDRX), which enhance battery life. NB-IoT devices, categorized under NB1 and NB2, support low data rates and efficient communication with maximum transport block sizes of 2536 bits for uplink and downlink, optimized for low latency applications. It uses Orthogonal Frequency Division Multiplexing (OFDM) for downlink and Single Carrier Frequency Division Multiple Access (SC-FDMA) for uplink, balancing efficiency and power usage. Mobility and positioning are enhanced through features like RRC Connection Re-establishment and observed time difference of arrival (OTDOA), ensuring stable connections and accurate device positioning. Additionally, NB-IoT supports multicast transmission and group messaging, beneficial for applications requiring firmware updates and group commands. These features make NB-IoT a versatile and efficient solution for various IoT applications, from smart metering and environmental monitoring to industrial automation and asset tracking^27^.

### 3GPP releases and the evolution of NB-IoT

The development of NB-IoT features across different 3GPP releases has significantly enhanced its capabilities, transforming it into a robust and versatile technology for IoT applications. Release 13 laid the foundation by introducing essential features such as coverage enhancement techniques for deeper indoor and rural areas, Power Saving Mode (PSM) for deep sleep states to conserve battery, and Extended Discontinuous Reception (eDRX) for balanced power savings and periodic communication. It also emphasized low device complexity and efficient signaling to reduce cost and power consumption. Release 14 built upon this framework by introducing several new functions designed to broaden NB-IoT’s scope and enhance its functionality. These included support for higher data rates, advanced positioning capabilities such as Observed Time Difference of Arrival (OTDOA) and enhanced Cell ID (eCID), and multicast functionality via Single-Cell Point-To-Multipoint (SC-PTM). This release also introduced the CAT NB-2 device category, which supports a higher Transport Block Size (TBS) of 2536 bits and a second HARQ process, as well as low-power user support with a maximum uplink transmission power of 14 dBm. These enhancements aimed to improve system capacity, flexibility, and energy efficiency, making NB-IoT suitable for a wider range of use cases, including mobile applications like smart parking and safety monitoring^28^.

Release 15 continued the evolution of NB-IoT, focusing on further performance enhancements and new features aimed at improving latency, power consumption, and network efficiency. Key additions included support for mobile-originated Early Data Transmission (EDT), which reduces latency and power usage by allowing small data transmissions during the random access procedure, and the Wake-Up Signal (WUS), which further lowers power consumption by reducing the need for devices to frequently check for network signals. Time Division Duplexing (TDD) was also introduced to optimize spectrum utilization for both uplink and downlink data^29^. Release 16 aimed to enhance NB-IoT features to support the growing number of devices and improve network operation efficiency. This included mobile-terminated EDT and group-specific WUS to reduce unnecessary wake-ups, and the Self-Organizing Network (SON) function to facilitate performance reporting and fault management. Additionally, Release 16 addressed issues related to NB-IoT’s coexistence with 5G NR, such as resource reservation, carrier placement, and synchronization^30^.

Releases 17 and 18 aim to further expand NB-IoT’s capabilities, supporting more complex use cases and improving efficiency. Planned enhancements include uplink and downlink support for 16-QAM, a new carrier selection scheme based on various factors, and the potential integration of NB-IoT with non-terrestrial networks. Other expected improvements include frequency hopping between carriers, cross-carrier scheduling, fine-grained channel quality reporting, escalated paging, very low user power class, and early termination of NPUSCH. These features across different 3GPP releases have collectively made NB-IoT a robust and versatile technology, capable of supporting a wide range of IoT applications with improved coverage, efficiency, and performance^31^^,^^32^.

### NB-IoT device operational modes and connection procedures

To achieve optimal performance and energy efficiency, NB-IoT employs a variety of operational modes and connection procedures tailored to different usage scenarios. These modes include Idle Mode, Connected Mode, Discontinuous Reception (DRX), Extended Discontinuous Reception (eDRX), and Power Saving Mode (PSM), each with distinct power consumption characteristics and network interaction protocols. The connection procedures in NB-IoT ensure that devices can efficiently transition between these modes, maintaining connectivity while minimizing energy use. These operational modes and connection procedures are crucial for developers and network operators aiming to maximize the efficiency and longevity of IoT devices deployed in diverse environments, from smart cities to remote agricultural fields. This section provides an in-depth exploration of the various NB-IoT device operational modes and the corresponding connection procedures, highlighting their significance in the broader context of IoT deployments.

#### Operational modes of NB-IoT

##### Idle mode

Idle Mode in NB-IoT represents a state where the device remains registered with the cellular network but is not actively involved in data transmission. Instead, it periodically listens to the paging channel, waiting for incoming data or signaling messages from the network. This intermittent monitoring allows the device to conserve battery power by minimizing active communication sessions. During Idle Mode, the NB-IoT device maintains its registration with the network, ensuring that it can quickly establish a connection when necessary. However, the device remains in a low-power state for the majority of the time, waking up only at predetermined intervals to check for any network activity. This approach is particularly beneficial for devices that do not require constant communication but need to remain reachable for occasional updates or commands.

##### Connected mode

Connected Mode is the active state of communication in NB-IoT, where the device establishes a continuous connection with the cellular network. In this mode, the device can send and receive real-time data, enabling applications requiring immediate responsiveness or continuous monitoring. During Connected Mode, the NB-IoT device maintains an open channel with the network, allowing for bidirectional communication. This mode is typically used to transmit sensor data, receive control commands, or engage in other interactive tasks. However, maintaining a constant connection requires higher power consumption compared to Idle Mode, as the device needs to remain fully powered and actively engaged in network activities.

##### Discontinuous reception (DRX) states

DRX is a power-saving mechanism employed by NB-IoT devices to reduce energy consumption while maintaining network connectivity. It consists of two primary states: DRX Active and DRX Inactive.

1. **DRX active state:** During DRX Active State, the device periodically wakes up from its low-power state to check for incoming data or signaling messages from the network. The device remains ready to receive information, ensuring timely responsiveness while conserving power by minimizing the duration of active network monitoring.
2. **DRX inactive state:** In contrast, DRX Inactive State allows the device to enter a deeper sleep mode, where network monitoring activities are suspended for an extended period. During this state, the device consumes minimal power, significantly extending battery life by reducing energy expenditure during idle periods.

##### Extended discontinuous reception (eDRX)

Extended Discontinuous Reception (eDRX) enhances the traditional DRX mechanism by allowing devices to remain in the inactive state for longer durations. This extended sleep cycle further reduces power consumption by prolonged periods of network inactivity, making it particularly beneficial for applications that demand infrequent data transmissions. eDRX enables NB-IoT devices to optimize energy usage by extending the intervals between network wake-ups, thereby maximizing the time spent in the low-power sleep state. This approach offers substantial energy savings without sacrificing connectivity, making it ideal for devices deployed in scenarios where frequent data transmission is not required.

##### Power saving mode (PSM)

Power Saving Mode (PSM) represents the deepest sleep state available to NB-IoT devices, where most components, including the radio module, are powered down to minimize power consumption. In this state, the device becomes unreachable by the network, effectively suspending all communication activities until a predefined wake-up event occurs. PSM is suitable for devices with sporadic data transmission requirements, such as smart meters or environmental sensors, where maintaining constant network connectivity is unnecessary. By entering a deep sleep state, NB-IoT devices can conserve energy over extended periods, significantly prolonging battery life and reducing the need for frequent recharging or battery replacement. By leveraging these operational modes effectively, NB-IoT devices can optimize power consumption while maintaining essential connectivity, ensuring efficient and sustainable IoT deployments across various applications and industries^33^.

**Fig. 2:**
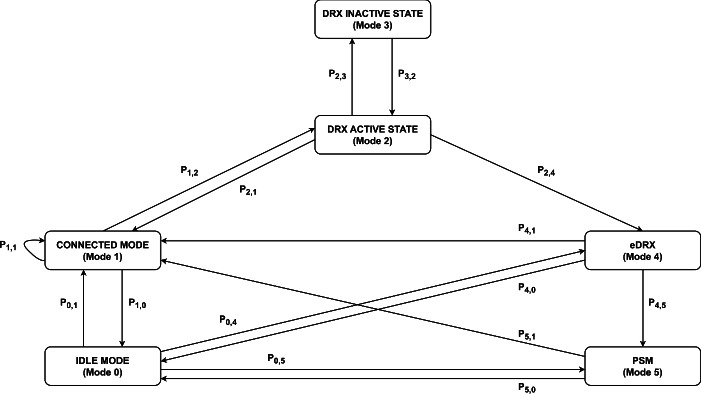
State transition diagram of NB-IoT device modes including Idle, Connected, DRX, eDRX, and PSM.

#### Connection procedures of NB-IoT

In the operational architecture of the Narrowband Internet of Things (NB-IoT), devices traverse through various operational modes, each intricately designed to balance connectivity and power efficiency. The journey typically begins in Idle Mode, where the device remains in a low-power state, periodically listening to the network’s paging channel. This mode serves as a power-saving mechanism, allowing the device to conserve energy while still staying registered on the network. Upon receiving a signaling message or triggering a network activity, the device transitions into Connected Mode, establishing an active connection with the network. In Connected Mode, the device engages in bidirectional communication, exchanging data packets with the network server. This mode ensures real-time data transmission and reception, vital for IoT applications requiring immediate responsiveness.

To further optimize power consumption, NB-IoT devices implement Discontinuous Reception (DRX) and Extended DRX (eDRX) states. DRX allows the device to alternate between active and inactive periods, waking up periodically to check for incoming data while spending the rest of the time in a low-power sleep mode. eDRX extends this concept by enabling longer sleep periods, reducing the frequency of wake-ups, and conserving more energy over extended durations. For scenarios where sporadic data transmission is acceptable, Power Saving Mode (PSM) offers the deepest sleep state. In PSM, the device powers down most of its components, including the radio module, to minimize energy consumption drastically. The device remains unreachable by the network until a pre-configured timer triggers a wake-up event, allowing it to re-establish connectivity and resume data transmission. Figure 2 illustrates the state diagram of the various operational modes of NB-IoT.

Through these meticulously crafted operational modes and states, NB-IoT devices achieve a delicate balance between connectivity and power efficiency, enabling prolonged battery life and uninterrupted operation for a diverse array of IoT applications^34^.

## Related work

In recent years, extensive research has been conducted to enhance energy efficiency and power-saving mechanisms in NB-IoT networks. Several studies have explored optimization techniques, reinforcement learning approaches, and energy-efficient scheduling mechanisms to improve network performance.

Hadjadj-Aoul and Ait-Chellouche proposed a deep reinforcement learning-based access control mechanism to mitigate congestion in NB-IoT networks. They modeled the access problem as a Markov decision process and used the Twin Delayed Deep Deterministic Policy Gradient (TD3) algorithm to optimize the Access Class Barring (ACB) mechanism. Unlike heuristic-based methods, their approach dynamically adjusted to network variations, even with incomplete system state information. Simulations showed superior performance over adaptive and PID-based techniques, maintaining optimal access attempts. Their study highlights reinforcement learning as a promising alternative for NB-IoT access control^35^.

Al Rabee et al. introduced an actor-critic reinforcement learning-based power allocation framework for energy harvesting (EH) NOMA relay-assisted mmWave networks to enhance energy efficiency and data throughput. Their two-phase approach first optimizes power allocation at an EH-capable source node using an actor-critic RL method, adapting to unpredictable EH and channel conditions. In the second phase, a NOMA-based mechanism assigns power levels to users for efficient relay transmission. Unlike conventional techniques that struggle with non-convex optimization, their method uses sequential convex approximation for better convergence. Simulations showed superior performance in maximizing data rates and improving energy efficiency, highlighting RL’s potential for resource allocation in next-generation networks^36^.

Lauridsen et al. conducted empirical power consumption measurements on early-generation NB-IoT devices to develop a battery lifetime estimation model. Their study provided the first publicly available dataset on real-world NB-IoT power usage across different operational states. Results showed that uplink transmissions at 23 dBm consumed 716 mW due to low power amplifier efficiency (37%), while receiving control/data channels used 213 mW. Idle-mode eDRX and PSM consumed 21 mW and 13 µW, respectively. Real-world power consumption exceeded 3GPP estimates, reducing battery life by 5.10% when PSM was applied. The study suggested firmware and hardware improvements to enhance future NB-IoT energy efficiency^37^.

Migabo et al. introduced the Energy-Efficient Adaptive Channel Coding (EEACC) scheme for NB-IoT to improve energy efficiency while maintaining network reliability. This two-dimensional approach dynamically selects the optimal channel coding scheme based on real-time channel conditions classified as bad, medium, or good, using periodic Block Error Rate (BLER) assessments. EEACC also reduces transmission repetitions by leveraging successful transmission probabilities, ensuring efficient resource use. Simulations showed that EEACC outperforms existing Narrowband Link Adaptation (NBLA) techniques in energy efficiency, reliability, scalability, and latency. Its resilience to channel impairments makes it ideal for energy-constrained IoT applications, with future validation planned for smart water metering and further theoretical optimization^38^.

Barbauzx et al. developed an analytical model to evaluate the balance between capacity and energy efficiency in NB-IoT systems, with a focus on battery life. Using M/D/H/K queues, their model assessed energy performance across different coverage distributions, payload sizes, and communication rates. Comparison with 3GPP results confirmed the models accuracy for single-terminal cells. Their analysis revealed that Early Data Transmission (EDT) not only improves latency and connection density but also enhances energy efficiency. However, higher loads in multi-terminal cells negatively impact battery life due to control channel demodulation. The authors proposed an efficient solution that extends battery life without modifying standard communication modules, making their model a valuable tool for optimizing NB-IoT energy efficiency^39^.

Khan and Alam developed an empirical model to evaluate the baseline energy consumption of NB-IoT radio transceivers, focusing on the Radio Resource Control (RRC) protocol. Using two commercial NB-IoT boards and test networks from two mobile operators, they collected data to create an accurate energy consumption model. Their profiling of the BG96 NB-IoT module showed evaluation errors between 0.33 and 15.38%, confirming the models reliability. This work fills a gap in energy profiling literature and serves as a benchmark for optimizing NB-IoT battery life. Future research will explore energy-saving strategies tailored to specific application requirements using this model^40^.

Manzar et al. investigated downlink (DL) packet reception energy consumption in NB-IoT and proposed a Particle Swarm Optimization (PSO)-based strategy to enhance energy efficiency. They analyzed key parameters such as transport block size, repetition count, and segmentation, optimizing factors like received power, sub-frames, and MAC header length. Their results showed an 84.98% energy reduction when optimizing PRX and HRLCMAC together and 61.07% when optimizing PRX and NSF. The study demonstrated PSOs potential for improving NB-IoT energy efficiency, with applications in smart homes, vehicles, and grids. Further enhancements could be achieved by integrating low-energy modulation and optimized MAC protocols^41^.

Andres-Maldonado et al. developed and validated an analytical energy consumption model for NB-IoT devices, aimed at improving energy management in Low-Power Wide-Area (LPWA) networks. Using a six-state Markov chain, their model estimated average energy consumption and latency for periodic uplink reporting. Experiments with two commercial NB-IoT devices connected to a base station emulator validated the model across scheduling, coverage extension, and single subcarrier configurations, with a maximum error of 21%. Their findings showed that NB-IoT UEs can achieve a 10 years battery life and 10-second latency under optimal conditions. This study contributes to energy-efficient strategies for future LPWA applications^42^.

Di Lecce et al. investigated cooperative relaying techniques to enhance energy efficiency in NB-IoT networks, aiming for further optimization despite its low power consumption. They proposed an optimal relay selection algorithm to minimize energy use within a cell and introduced a greedy algorithm that achieved near-optimal performance with lower computational complexity. Simulations showed that cooperative relaying reduced energy consumption by up to 30%, with the greedy algorithm consuming only 10% more than the optimal strategy. Their findings highlight cooperative relaying as an effective energy-saving approach. Future research will explore throughput, delays, and advanced power control mechanisms for further optimization^43^.

Jiang et al. proposed a Cooperative Multi-Agent Deep Q-Learning (CMA-DQN) approach to optimize multi-group NB-IoT networks, addressing configuration challenges without prior traffic statistics. In this model, Deep Q-Network (DQN) agents independently control configuration variables and are cooperatively trained based on transmission feedback. Compared to heuristic-based load estimation (LE-URC), CMA-DQN significantly outperformed it, especially in heavy traffic, by dynamically adjusting repetition values to optimize resource allocation. This improved Random Access Opportunities (RAOs) and reduced collisions. Their results highlight CMA-DQN as an effective solution for managing scarce resources and enhancing NB-IoT performance under varying traffic conditions^44^.

Jiang et al. developed Q-learning-based methods to optimize uplink resource configurations in NB-IoT networks, maximizing served IoT devices per Transmission Time Interval (TTI). They introduced tabular Q-learning (tabular-Q), Linear Approximation Q-learning (LA-Q), and Deep Q-learning (DQN), all of which outperformed heuristic-based load estimation (LE-URC) approaches. LA-Q and DQN achieved similar performance to tabular-Q but required less training time. To handle high-dimensional configurations, they extended LA-Q and DQN with Action Aggregation (AA-LA-Q, AA-DQN), improving convergence. Additionally, Cooperative Multi-Agent DQN (CMA-DQN) was introduced for parallel sub-task optimization, showing superior efficiency. Their findings highlight Q-learning as a robust solution for real-time NB-IoT resource allocation^45^.

Michelinakis et al. conducted an empirical study on NB-IoT energy consumption, analyzing the impact of configuration parameters on efficiency. Using measurements from two NB-IoT boards and two European operators, they found that while NB-IoT is marketed as plug-and-play, energy efficiency depends on proper configuration. Paging intervals in the connected state significantly affected power use, with some operators misconfiguring these settings. Packet size and signal quality had minimal impact unless signal strength was very poor. Adjustments like enabling RAI and eDRX led to major energy savings. Their findings emphasize the role of module settings, operator configurations, and energy-saving mechanisms in battery life, suggesting further research on protocol tuning for improved efficiency^46^.

Rastogi et al. proposed a semi-Markov-based energy-saving model for NB-IoT devices, introducing an Auxiliary State in the DRX mechanism to reduce power consumption, especially for small data transmissions. Unlike traditional approaches, this method optimizes energy use by minimizing unnecessary activity when data packets are minimal. Evaluations showed power-saving improvements of up to 97.1 and 98.25% by adjusting eDRX and PSM timers. The model effectively conserved energy without significant delay increases across various data arrival rates. Their findings highlight the potential of integrating additional states into NB-IoT mechanisms for better energy efficiency, with future research focusing on further parameter optimization^47^.

Zhang et al. proposed a power control scheme to enhance energy efficiency (EE) in the Narrowband Physical Uplink Shared Channel (NPUSCH) of NB-IoT, addressing interference from its non-orthogonality with NPRACH. They introduced guard bands to mitigate interference and formulated an EE optimization problem considering circuit power consumption and minimum data rates. Using fractional programming, they developed an iterative power control algorithm that quickly converged to near-optimal solutions. Simulations showed significant EE improvements with guard bands, especially for low data rate communications. However, the trade-off between EE and spectral efficiency (SE) requires further exploration for high-data-rate applications^48^.

Sultania et al. developed an energy consumption model for NB-IoT devices using Power Saving Mode (PSM) and Extended Discontinuous Reception (eDRX) to evaluate energy efficiency in large-scale IoT deployments. Based on a Poisson arrival process, their model showed an average error of 11.82% compared to NS-3 simulations. Results indicated that with a 5 Wh battery and optimized PSM/eDRX settings, NB-IoT devices could last over 12 years with one packet transmission per day. However, small Idle state timers increased energy consumption by 3 to 7 times. Their findings emphasize the importance of proper power-saving configurations for extended battery life in IoT applications like shared bicycle tracking^49^.

Navarro et al. conducted a comparative study on the energy consumption of various communication protocols MQTT, TCP, UDP, and LwM2M used in NB-IoT applications with the BG96 module. They found that UDP had the lowest energy consumption, especially with frequent transmissions, while MQTT was the most cost-effective for feature-rich IoT applications. Payload size (10100 bytes) had minimal impact on energy use, allowing flexible data transmission. Their results emphasize that protocol choice should align with system requirements, with UDP suited for low-power needs and MQTT for cost-efficient solutions. Their methodology provides a foundation for further optimizing energy consumption in IoT communication protocols^50^.

Elhaddad et al. evaluated the energy consumption of three NB-IoT modules under simulated LTE network conditions, analyzing factors such as T3324, T3412, uplink transmit power, and SIB message parameters. They developed a data traffic-dependent energy model to estimate battery lifetime under different communication scenarios, including periodic UDP uplink transmissions. Their findings showed that energy consumption per bit varied with NPUSCH repetitions, highlighting the impact of transmission periods on power usage. Optimizing power amplifier (PA) design and hardware architecture could further improve energy efficiency. The study suggests that firmware, hardware, and network optimizations will enhance future NB-IoT device battery life^51^.

Abbas et al. studied NB-IoT energy consumption, analyzing the impact of tunable and non-tunable parameters on efficiency. They found that enabling full Discontinuous Reception (DRX), especially connected-mode DRX (cDRX), could cut energy use by up to 50% over 10 years. The RRC inactivity timer played a crucial role, while CoAP retransmission timers and eDRX cycles had minimal impact. Traffic intensity and burstiness significantly influenced energy usage, with lower-intensity data bursts reducing power consumption. Their study provided guidelines for optimizing the NB-IoT protocol stack to meet the 3GPP 10-year battery life target. Future research will compare full vs. partial DRX support and validate findings through real-world NB-IoT testbed measurements^52^.

Chen et al. proposed an energy-efficient multi-hop LoRa broadcasting scheme (FLBS) for IoT networks, optimizing transmission energy consumption and large-scale data distribution. Using reinforcement learning for optimal relay selection, FLBS reduced communication time by 87.4% and saved 12.61% more energy than traditional methods. It proved highly effective for small-scale IoT applications like remote upgrades in circular areas but faced challenges in large regions with limited channels. Future work will extend FLBS to larger areas, integrate caching, explore device-to-device (D2D) communication, and apply it to smart city and power delivery systems to further enhance energy efficiency^53^.

Yu and Lo studied energy-efficient non-anchor channel allocation in NB-IoT cellular networks, identifying that increasing non-anchor channels can sometimes raise device energy consumption. Unlike traditional allocation problems, this exhibits a non-convex property. To address this, they developed a dynamic programming algorithm to determine the optimal number of non-anchor channels per base station, minimizing energy use. They also proposed an energy-efficient channel reuse algorithm, reducing energy consumption by 66% compared to baseline methods. Their findings highlight the need for careful channel allocation to prevent unnecessary power consumption in NB-IoT transmissions^54^.

Yu and Wu investigated energy-efficient scheduling for search-space periods in NB-IoT, aiming to reduce blind decoding (BD) and idle time. Since base stations can only schedule devices with the same search-space period per subframe, resource allocation is limited. They proposed an algorithm to optimize search-space periods and a scheduling method that reduces BD and idle time while meeting data demands. Their approach lowered energy consumption by 77% compared to baseline methods. Findings showed that reducing search-space periods and DCI repetitions had a greater impact on base station energy use than on devices. Future work will explore multiple non-anchor channels and base stations for further optimization^55^.

Liang et al. tackled energy-efficient uplink resource unit (RU) scheduling for ultra-reliable NB-IoT communications, modeling it as an NP-complete optimization problem. They proposed a two-phase scheduling scheme: the first phase optimizes default transmission settings to minimize energy use while meeting QoS requirements, while the second phase balances transmission urgency and flexibility to ensure delay constraints. Simulations showed that their method effectively reduced energy consumption while serving more devices with guaranteed QoS. Their findings demonstrate NB-IoT’s capability to support large-scale IoT applications with minimal energy usage, making it a strong candidate for energy-efficient 5G communications^56^.

Zholamanov et al. proposed an enhanced reinforcement learning algorithm, Double Deep Q-Network with Prioritized Experience Replay (DDQN-PER), to optimize energy consumption (EC) and packet delivery ratio (PDR) in LoRa wireless networks. Their method selects optimal transmission parameters, such as spreading factor (SF) and transmission power (TP), to minimize energy use while maximizing PDR. Simulations showed a 17.2% PDR improvement over Adaptive Data Rate (ADR) and a 6.2 to 8.11% boost over other RL-based methods. DDQN-PER excelled in large-scale networks (1000 devices) and maintained performance in obstacle-prone environments. Future research will validate the algorithm in real LoRaWAN networks, explore mobile node adaptation, and integrate it with other communication protocols for greater efficiency^57^.

Bortnik et al. developed a machine learning (ML)-based method to estimate NB-IoT device energy consumption using statistical modem data instead of additional circuitry. They created a labeled dataset using an NB-IoT module with an onboard current measurement circuit, analyzing parameters like radio channel quality, transmission power, and TX/RX time. Feature selection showed strong correlations between energy consumption and temporal parameters. Among 11 ML models evaluated, Decision Tree Regression (DTR), Gradient Boosting (GBR), XGBoost (XGBR), and Polynomial Regression (PR) achieved up to 93.8% accuracy with minimal memory use (as low as 3 KB). Future research will explore advanced ML models, improved feature selection, and on-device self-estimation for energy efficiency^58^.

Lingala et al. compared Power Saving Mode (PSM) and Power Down Mode (PDM) in NB-IoT modems using a Quectel modem. While PDM had lower current consumption for over 95% of the time, PSM proved more energy-efficient overall, considering active, idle, and sleep periods. PDM introduced additional signaling overhead and delays in uplink/downlink transmissions, reducing its advantages. PSM consistently outperformed PDM in most scenarios, except when base stations provided lower-than-required T3412 timer values. The study concluded that PSM is the preferred mode for NB-IoT, offering a better balance between power savings and communication efficiency^59^.

Caso et al. conducted a large-scale data-driven analysis of the Random Access (RA) procedure in NB-IoT networks, examining the impact of deployment, radio coverage, and operator configurations. While RA generally met performance requirements, increasing connectivity and scenario variability posed optimization challenges. They proposed a Machine Learning (ML)-based enhancement, using radio conditions like RSRP, SINR, and RSRQ to predict RA success and delay with high accuracy. Their approach optimized RA configurations, reducing power consumption by at least 50%. Future work will explore implementation in dynamic environments and advanced system scenarios for further optimization^60^.

Lukic et al. conducted a real-world evaluation of NB-IoT module energy consumption using a custom-designed high-resolution data collection platform. Their study analyzed energy usage across different transmission phases, highlighting the impact of both device and network-side configurations. Experiments with a mobile operator revealed significant variations in energy consumption depending on UE and eNB settings. Future plans include scaling the study to 100 NB-IoT nodes to gather extensive data under various configurations. Their findings provide valuable insights into real-world NB-IoT energy efficiency, crucial for maximizing battery life in large-scale deployments^61^.

Zhao et al. proposed an intelligent NB-IoT-based street lighting system with an energy-saving algorithm to reduce energy consumption, maintenance costs, and operational complexity. The system integrates a cloud server, remote monitoring, and streetlight control terminals, using NB-IoT and Power Line Carrier (PLC) communication for intelligent local and remote control. It adjusts brightness based on ambient light and vehicle speeds, enabling on-demand lighting to save energy. Additionally, it supports environmental monitoring, fault alarms, and abnormal protection. The system improves adaptability, cost efficiency, and real-time responsiveness, making it a promising solution for future smart city infrastructure^62^.

Kim et al. proposed a multi-agent reinforcement learning (MARL) framework, MAQ-OCB, to optimize energy efficiency (EE) and minimize user outages in ultra-dense small cell networks. Using distributed Q-learning for outage-aware cell breathing, the framework reduces network energy consumption while maintaining QoS in 6G wireless networks. Simulations showed MAQ-OCB outperformed traditional algorithms like No TPC, On-Off, and centralized Q-learning (C-OCB). Two variations were tested: one using neighboring small cell base station (SBS) state information and another relying only on its own state. Results confirmed MAQ-OCB’s effectiveness in improving EE and reducing outages, demonstrating its potential for energy-efficient 6G networks^63^.

Alamu et al. reviewed machine learning (ML) applications in energy harvesting (EH) IoT networks, focusing on challenges from stochastic energy sources and wireless fading channels. They explored ML techniques such as reinforcement learning (RL), deep learning (DL), and deep reinforcement learning (DRL) for optimizing energy usage. While RL adapts well to environmental changes, it struggles with large state-action spaces in massive IoT deployments. DRL offers better data processing but requires energy-efficient optimization for practical use. The study highlighted the need for lightweight DRL models to support EH in large-scale IoT networks, particularly for future 6G applications^64^.

Guo and Xiang proposed a multi-agent reinforcement learning (MARL) framework to optimize energy efficiency in NB-IoT networks by improving power ramping and preamble allocation. Traditional random preamble allocation in LTE lacks efficiency for large-scale IoT deployments. Their joint optimization approach integrates power ramping and preamble selection, enhancing energy efficiency and random access (RA) success probability. Using a Win-or-Learn-Fast Policy Hill-Climbing (WoLF-PHC) algorithm with a simplified “stateless” modification, simulations demonstrated significant energy savings. Future work will incorporate Power Saving Mode (PSM), coverage enhancement (CE) classes, and state variables like RSRP to further refine optimization^65^.

Chen et al. proposed an energy-efficient LoRa broadcasting scheme, FLBS, for IoT applications like remote upgrades in circular areas. By combining LoRa protocols with multi-hop technology, the scheme optimizes relay selection and transmission power to reduce energy consumption. Using a reinforcement learning-based algorithm, FLBS outperformed traditional methods in energy savings. The study emphasized the importance of considering actual LoRa hardware parameters and environmental factors. Future research will explore caching, device-to-device (D2D) communication, and integrating LoRa mesh to enhance scalability and applicability in complex IoT scenarios^66^.

Haridas et al. examined the use of energy-harvesting technologies to extend NB-IoT device battery life in smart home applications. Their analysis of energy consumption across coverage classes revealed discrepancies between actual and expected 10-year lifespans. They explored ambient energy sources for harvesting, showing that, in ideal conditions, perpetual operation was possible but highly dependent on energy availability. Key challenges included managing unpredictable energy sources and optimizing long-term sustainability. Their findings highlight the potential of energy harvesting for improving NB-IoT efficiency, with future work focusing on overcoming integration challenges for reliable IoT applications^67^.

Chang et al. optimized NB-IoT power consumption using adaptive radio access (RA) strategies, focusing on enhanced coverage levels (ECLs). Through field measurements on two testbeds, they identified inefficiencies in ECL selection and proposed an adaptive RA approach incorporating predictive ECL selection and opportunistic packet transmission. Their method reduced UE power consumption by up to 36% while maintaining block error rate (BLER) performance. The study emphasized ECL selection’s role in improving energy efficiency without compromising reliability. Future work will refine uplink quality predictions and optimize ECL selection from both UE and eNodeB perspectives^68^.

Sultania et al. developed an analytical model to evaluate NB-IoT power consumption and downlink (DL) latency using Power Saving Mode (PSM) and extended Discontinuous Reception (eDRX). Based on a Markov chain, the model accurately predicted energy consumption and latency, achieving over 91% accuracy compared to ns-3 simulations. Their multi-objective Pareto analysis identified optimal parameter configurations, favoring smaller timer values for low-latency or infrequent uplink (UL) traffic scenarios. Future research will explore additional power-saving techniques, such as Release Assistance Indication (RAI), Wake-up signals, and Early Data Transmission, to further enhance NB-IoT energy efficiency^69^.

Jorke et al. analyzed the power consumption of NB-IoT and eMTC in smart city environments, comparing data rate, battery life, latency, and spectral efficiency under different coverage conditions. Their study found that eMTC outperformed NB-IoT in moderate conditions (144 dB coupling loss) with a 4% longer battery life and higher data rates. However, in extreme conditions (164 dB coupling loss), NB-IoT provided an 18% longer battery life due to reduced transmission repetitions. While eMTC performed better at 155 dB or lower, NB-IoT’s superior spectral efficiency and lower bandwidth needs make it ideal for large-scale IoT deployments^70^.

Duhovnikov et al. evaluated the feasibility of NB-IoT for low-power aircraft applications, conducting experiments with a Sodaq NB-IoT module on private and commercial networks. Their findings showed that optimizing Power Saving Mode (PSM) could extend battery life for several years, but configuration and hardware design play a crucial role in aviation use cases. While NB-IoT demonstrated promise for certain applications, 5G was deemed necessary for more demanding aviation needs. The study emphasized optimizing peripheral energy consumption and extending transmission cycles to improve battery life, with future research focusing on further enhancements for aviation scenarios^71^.

Lee and Lee proposed a Prediction-Based Energy Saving Mechanism (PBESM) to enhance NB-IoT uplink transmission efficiency by reducing energy consumption. PBESM includes a deep packet inspection-based network architecture to predict uplink packet occurrences and an algorithm that optimizes scheduling requests by pre-assigning radio resources. This reduces random access attempts, lowering transmission energy use by up to 34%. Additionally, PBESM improved session active time by 16% without requiring hardware modifications on IoT devices. Future research will integrate software-defined networking for better packet inspection and explore contention resolution in multi-user scenarios to enhance efficiency^21^.

Alobaidy and Singh conducted a real-world evaluation of NB-IoT performance in Malaysia, analyzing coverage, path loss, packet delivery rate (PDR), latency, and power consumption. NB-IoT achieved a 91.76% PDR, supporting high data rates even with low signal quality, but latency variations significantly impacted battery efficiency. Compared to LoRaWAN and Sigfox, NB-IoT had a much shorter battery life 344.9 days versus 1608.9 and 1527.6 days, respectively. While NB-IoT excelled in data rate and coverage, its power consumption was higher than expected. The study emphasized optimizing power management and deployment strategies for better efficiency and highlighted their measurement platform as a useful tool for IoT network tracking^72^.

Alkhayyal and Mostafa conducted a systematic literature review on the role of machine learning (ML) and artificial intelligence (AI) in enhancing LoRaWAN energy efficiency and performance for IoT applications. Their review highlighted the effectiveness of deep reinforcement learning (DRL) and supervised learning in optimizing resource allocation, network stability, and energy consumption. Key factors such as Spreading Factor (SF), bandwidth (BW), and coding rate (CR) were identified as crucial for balancing communication range, data rate, and power efficiency. The study emphasized the need for adaptive ML-based algorithms to dynamically adjust network parameters. Future research will focus on real-time adaptive systems and cross-layer optimization for improved network performance^73^.

Nauman et al. investigated Intelligent Device-to-Device (I-D2D) communication to optimize data delivery and energy efficiency in NB-IoT, particularly for delay-sensitive applications like healthcare IoT. They addressed the high power consumption caused by repeated control and data transmissions between NB-IoT User Equipment (UE) and base stations. Their proposed two-hop D2D communication model reduced transmission repetitions, improving efficiency. Relay selection was formulated as a Multi-Armed Bandit (MAB) problem and solved using a Reinforcement Learning (RL) approach. Simulations showed that I-D2D improved Packet Delivery Ratio (PDR) and reduced End-to-End Delay (EED). Future work will focus on large-scale deployment and real-world integration into IoT networks^74^.

Pei, Zhang, and Li proposed an energy-saving mechanism for NB-IoT based on extended discontinuous reception (eDRX), focusing on power consumption and access delay. They developed a Markov model to analyze NB-IoT device states, incorporating the random access process often overlooked in energy calculations. Their findings showed that backoff time after access failures significantly impacts energy consumption and delay. By linearly increasing backoff time, they reduced variations in access delays and improved energy efficiency. This study provides valuable insights into optimizing NB-IoT power management, particularly in scenarios with frequent network access attempts^75^.

Bali et al. explored the energy efficiency of NB-IoT in smart applications, emphasizing its role in reducing IoT energy consumption and carbon footprints. They highlighted the integration of Green IoT with NB-IoT as a promising approach, particularly for large-scale applications like smart agriculture. NB-IoT’s low power usage, massive connectivity, and strong indoor coverage make it well-suited for sustainable IoT solutions. They proposed a green NB-IoT model for agriculture to promote energy-efficient technologies. While NB-IoT is cost-effective and reliable, challenges remain in further optimizing energy efficiency for large-scale deployments, necessitating continued research^18^.

Anbazhagan and Mugelan proposed an energy-saving technique for NB-IoT, integrating a Proxy state and enhanced Release Assistance Indication (ERAI) within a semi-Markov framework. This approach optimizes the Discontinuous Reception (DRX) mechanism by reducing unnecessary wake-ups, significantly improving the Power Saving Factor (PSF). Their method achieved up to 99.4% energy savings with optimized eDRX durations and 99.9% with optimized PSM settings, extending device battery life for low-data applications. Future work will refine the semi-Markov model, validate it in real-world scenarios, and explore trade-offs between energy efficiency and communication delays, with potential adaptation for other LPWAN technologies^76^.

Anbazhagan and Mugelan introduced a Soft Actor-Critic (SAC) reinforcement learning algorithm to optimize resource allocation in NB-IoT networks, tackling challenges like dynamic user demands and variable channel conditions. SAC outperformed traditional methods like DQN and PPO, improving energy efficiency by 10.25%, throughput by 214.98%, and fairness (Jain’s index) by 614.46%. It also enhanced recovery time and marginally improved latency, making it ideal for energy-efficient, low-latency applications. SAC demonstrated scalability across urban, industrial, and rural IoT deployments, proving to be a robust solution for optimizing NB-IoT resource allocation and network performance^77^.

### Technical gaps and research motivation

Despite significant advancements in energy-efficient NB-IoT systems, several challenges remain. Most existing studies focus on either static optimization techniques or isolated power-saving mechanisms, lacking a comprehensive and adaptive approach. While considerable attention has been given to downlink optimization, uplink energy efficiency essential for prolonged device operation has been largely neglected. Additionally, while reinforcement learning has been explored for resource allocation, its integration with power-saving mechanisms and intelligent decision-making models is still in its early stages. Empirical studies also reveal inconsistencies between theoretical models and real-world energy consumption, highlighting the need for adaptive power control strategies. A major limitation of current approaches is the lack of dynamic power-saving mode switching based on real-time network conditions. Most existing mechanisms operate under fixed configurations, resulting in inefficient energy utilization. Furthermore, although cooperative relaying and multi-hop strategies have been investigated in other domains, their potential for enhancing power-saving in NB-IoT remains largely unexplored.

To address these challenges, this research introduces an adaptive power-saving mode control framework based on Soft Actor-Critic (SAC) reinforcement learning. Unlike conventional methods, this approach dynamically adjusts power-saving modes in response to changing network conditions, ensuring optimal energy efficiency while maintaining Quality of Service (QoS). By bridging the gap between theoretical energy models and practical deployment constraints, this framework offers a more effective and scalable solution. By integrating reinforcement learning with established power-saving modes such as Power Saving Mode (PSM) and extended Discontinuous Reception (eDRX), this research provides a flexible and adaptive power management strategy. Unlike traditional methods the proposed approach ensures a real-time balance between energy efficiency and service quality, making it particularly well-suited for large-scale NB-IoT deployments.

Traditional static power-saving strategies in NB-IoT, such as fixed DRX (Discontinuous Reception) or PSM (Power Saving Mode) configurations, rely on pre-defined timers and thresholds or deterministic scheduling rules that do not respond to dynamic changes in network traffic, signal quality, or application requirements. While such rule-based approaches are simple to implement and computationally inexpensive, they lack the flexibility to adapt in real time. As a result, they often lead to suboptimal energy consumption, increased latency, or reduced reliability under fluctuating conditions.

In contrast, the proposed Soft Actor-Critic (SAC)-based power management approach continuously interacts with the environment and learns to adapt its mode-switching policy based on evolving system states. This adaptability allows the SAC agent to balance energy efficiency and transmission reliability more effectively than static methods. Given the inherently time-varying and device-specific nature of NB-IoT deployments, static models were deemed unsuitable for simulation in this context. Instead, our focus was on benchmarking against dynamic deep reinforcement learning algorithms (DQN and PPO), which offer a more realistic performance baseline for intelligent control in heterogeneous and uncertain IoT environments.

This research leverages the Soft Actor-Critic (SAC) algorithm to improve power management in NB-IoT networks, offering significant advancements over existing methods. Unlike traditional approaches, SAC dynamically adjusts power-saving modes based on real-time network conditions, enhancing adaptability and efficiency. SAC stands out from other reinforcement learning algorithms like Proximal Policy Optimization (PPO), and Deep Q-Networks (DQN), through its handling of continuous action spaces and entropy regularization, ensuring robust exploration and preventing premature convergence to suboptimal policies. Additionally, SAC’s off-policy learning and stochastic policy capabilities allow for efficient data utilization and adaptability to fluctuating network conditions, leading to more reliable and context-aware power-saving decisions. By addressing the limitations of existing solutions, the SAC-based approach significantly enhances power efficiency, network performance, and overall sustainability in NB-IoT networks, paving the way for more resilient and scalable IoT deployments.

## Methodology

### NB-IoT environment simulation

We implement a reinforcement learning framework for optimizing power-saving modes in a Narrowband Internet of Things (NB-IoT) network. This framework comprises several key components, each contributing to the simulation and optimization process.

Firstly, the *NB-IoT Environment class* encapsulates the environment in which the devices operate. It defines a state space with 17 dimensions, encompassing parameters such as signal strength, battery level, data rate, spectral efficiency, power consumption, resource utilization, energy efficiency, retransmission rate, latency, channel quality indicator (CQI), packet loss, queuing delay, mobility, and temperature. The action space consists of six distinct power-saving modes: Mode 0 (Idle Mode), Mode 1 (Connected Mode), Mode 2 (DRX Active State), Mode 3 (DRX Inactive State), Mode 4 (Extended DRX - eDRX), and Mode 5 (Power Saving Mode - PSM). The reset method initializes the state of each device with random values within standard ranges. The step method applies the selected action, updates the state, and computes a reward based on a weighted combination of the mentioned parameters, ensuring non-negative rewards and penalizing undesirable conditions like high retransmission rates, latency, packet loss, and queuing delay.

The *SAC Agent class* implements the Soft Actor-Critic (SAC) algorithm, a reinforcement learning technique. This agent includes Q-value networks (critics) and a policy network (actor). The critics evaluate state-action pairs, while the actor samples actions based on the current policy, providing a mean and log standard deviation for the actions. The SAC Agent undergoes training using experiences stored in a replay buffer, which consists of state transitions recorded during the simulation. Training involves updating the Q-value networks to minimize the loss between predicted and target Q-values and adjusting the policy network to maximize expected rewards. The agent also offers methods for saving and loading model weights, facilitating model persistence.

The *Centralized Controller class* utilizes the SAC Agent to select actions for multiple devices in a centralized manner. It prioritizes specific power-saving modes, including Mode 4 (eDRX) and Mode 5 (PSM), by adjusting action probabilities to favor these modes for improved power efficiency. During each training episode, the controller aggregates experiences from all devices, store them in a replay buffer, and trains the SAC Agent using this buffer. This centralized approach ensures coordinated optimization across all devices in the network.

The simulation and training loop initialize an environment with 10 devices and set up the SAC Agent and Centralized Controller. Over 1000 episodes, the environment is reset at the start of each episode, and actions are selected for all devices using the centralized controller. The resulting states, rewards, and done signals are recorded, and various metrics, including power consumption, signal strength, battery level, data rate, spectral efficiency, packet loss, queuing delay, CQI, latency, and energy efficiency, are monitored. The collected experiences are utilized to train the SAC Agent, and episode-wise metrics are aggregated and saved to an Excel file for detailed analysis. Additionally, the total rewards per episode are plotted to visualize the learning progress of the agent. Tables 1 and 2 presents the simulation parameters for the NB-IoT environment and the SAC algorithm, detailing the key values and configurations for network, environment, and algorithm settings. These parameters define the operation of both the NB-IoT device modes and the reinforcement learning setup.

To evaluate the performance of the proposed Soft Actor-Critic (SAC)-based power management strategy, we compare it against two standard deep reinforcement learning algorithms: Deep Q-Network (DQN) and Proximal Policy Optimization (PPO). DQN represents value-based learning, where Q-values guide action selection, while PPO is a state-of-the-art policy gradient method that balances exploration and stability. These models are not static or rule-based, but serve as dynamic, learning-based baselines to measure how SAC’s entropy-regularized learning improves long-term energy efficiency and reward optimization in NB-IoT environments.

**Table 1:** NB-IoT environment parameters.

Parameter	Description	Value/Configuration
Network type	Type of IoT network used for simulation	NB-IoT
Channel model	Channel model applied during simulation	Rayleigh Fading, Free-Space
Environment	Simulation environment	Python (Gym), MATLAB
Training episodes	Number of training episodes	1000
Time slots	Time slots for communication	10ms per slot
Frequency band	The frequency range used for NB-IoT	800 MHz
Data rate	The communication data rate for the simulation	250 kbps
Transmission power	Transmission power for devices	14 dBm
Noise power	Noise level in the environment	-104 dBm
Channel bandwidth	Channel bandwidth for communication	200 kHz
Idle mode timer	The device remains registered with the network, periodically waking up to check for activity.	10 s
Connected mode timer	The device maintains continuous communication with the network for active data transmission.	30 s
DRX active state timer	The device wakes up periodically to check for incoming data while conserving power.	10 s
DRX inactive state timer	The device enters a deeper sleep state, minimizing power consumption by suspending network monitoring.	180 s
Extended DRX (eDRX) timer	The device stays inactive for longer intervals, saving power while maintaining low connectivity.	180 s
Power saving mode (PSM) timer	The device enters deep sleep, suspending all communication until a wake-up event occurs.	3600 s

**Table 2:** SAC algorithm parameters.

Parameter	Description	Value/Configuration
Learning rate	Learning rate for the SAC agent	0.0003
Discount factor ($\gamma$)	Discount factor used for future rewards	0.99
Batch size	Number of experiences in each batch	128
Replay buffer size	Size of the experience replay buffer	1,000,000
Update frequency	Frequency of updating the policy	50 steps
Hidden layer sizes	Number of units in each hidden layer of the neural network	[256, 256]
Policy network type	Architecture of the policy network	MLP (Multilayer Perceptron)
Q-Network type	Architecture of the Q-network	MLP
Action space	Type of action space	Continuous
State space	Type of state space	Continuous
Entropy coefficient ($\alpha$)	Weight of the entropy term in the objective function	0.2
Target network update rate	The rate at which target networks are updated	0.005
Exploration strategy	The method used to encourage exploration	Gaussian Noise
Number of actors	Number of parallel actors used in training	16

### Soft actor-critic (SAC) algorithm

Soft Actor-Critic (SAC) is a sophisticated off-policy reinforcement learning algorithm tailored for continuous action spaces, aiming to develop a stochastic policy that maximizes cumulative rewards while ensuring policy entropy regularization to foster exploration and robustness. In the context of Intelligent Power Management in NB-IoT networks, SAC dynamically selects power-saving modes for devices based on observed states and rewards. The SAC algorithm encompasses a soft Q-learning framework, comprising two crucial components: the actor network and the critic network.

The actor network approximates the policy function $\pi (s)$, generating a Gaussian distribution with mean $\mu (s)$ and standard deviation $\sigma (s)$, dictating the probability distribution over actions given states:

1 $$\begin{aligned} \pi (a \mid s)=\mathcal {N}(a \mid \mu (s), \sigma (s)) \end{aligned}$$

Meanwhile, the critic network estimates the state-action value function *Q*(*s*, *a*), denoted as $Q_\phi (s, a)$, evaluating the quality of taking action *a* in state *s* :

2 $$\begin{aligned} Q(s, a) \approx Q_\phi (s, a) \end{aligned}$$

To delve into the mathematical underpinnings, the soft Bellman backup is employed to update the critic network. It calculates the target value $y_t$ as the sum of the immediate reward $r_t$ and the expected future reward, balancing exploration and exploitation through entropy regularization:

3 $$\begin{aligned} y_t=r_t+\gamma \mathbb {E}_{s^{\prime } \sim p}\left[ Q^{\text{ target } }\left( s^{\prime }, \pi _\theta \left( s^{\prime }\right) \right) -\alpha \log \pi _\theta \left( a \mid s^{\prime }\right) \right] \end{aligned}$$

where:

- $y_t$ is the target value.
- $r_t$ is the reward at time step *t*.
- $s^{\prime }$ is the next state sampled from the environment.
- $Q^{\text{ target } }$ denotes the parameters of the target critic network.
- $\pi _\theta$ denotes the parameters of the actor network.
- $\alpha$ is the temperature parameter for entropy regularization.

The actor’s objective is to maximize the expected reward under the current policy while simultaneously maximizing policy entropy. This involves optimizing the parameters $\theta$ of the actor network by ascending the gradient of the actor objective function, $J(\theta )$, computed as the expectation over states and actions:

4 $$\begin{aligned} J(\theta )=\mathbb {E}_{s \sim \rho ^\pi }\left[ \mathbb {E}_{a \sim \pi _\theta }\left[ Q_\phi (s, a)-\alpha \log \pi _\theta (a \mid s)\right] \right] \end{aligned}$$

During training, SAC utilizes off-policy data stored in an experience replay buffer, updating the actor and critic networks iteratively using stochastic gradient descent. The critic network parameters $\phi$ are updated to minimize the soft Bellman backup loss, while the temperature parameter $\alpha$ for entropy regularization is adjusted to maintain a balance between exploration and exploitation. In the context of NB-IoT power management, a centralized controller facilitates coordinated power-saving mode selection for multiple devices, leveraging the learned policy from SAC to ensure optimized network behavior.

Soft Actor-Critic (SAC) offers a comprehensive framework for intelligent power management in NB-IoT networks, integrating entropy regularization to encourage exploration while efficiently learning from off-policy data. By incorporating sophisticated algorithms and iterative updates, SAC enables sustainable and energy-efficient operation of NB-IoT networks in real-world scenarios, paving the way for adaptive and responsive power management solutions.

### Reward function

In the Soft Actor-Critic (SAC) algorithm for NB-loT power management, the reward function is designed to optimize a comprehensive set of performance metrics while balancing power consumption. Key metrics considered in the reward function include signal strength (RSSI), battery level, data rate, energy consumption, latency, packet error rate, and throughput, among others. The reward function $R_t$ is formulated to ensure the device operates efficiently across various dimensions critical to its performance. Signal strength, measured by RSSI, is crucial for maintaining reliable communication and reducing retransmissions. Battery level is monitored to prolong the operational life of NB-loT devices, essential for deployments in remote locations. The data rate ensures efficient and timely data transmission, vital for applications requiring real-time data. Energy consumption is minimized to promote the use of power-saving modes, thereby extending battery life, and hence, it is included with a negative sign in the reward function to incentivize lower energy usage. Latency is considered to ensure quick response times, critical for time-sensitive applications. Additionally, metrics such as packet error rate and throughput are included to maintain communication reliability and optimize data flow. The packet error rate is also included with a negative sign to encourage minimizing errors. The reward function $R_t$ is expressed as a weighted sum of these metrics:

5 $$\begin{aligned} \begin{aligned} R_t =&\ w_{\text {RSSI}} \cdot \text {RSSI}_t + w_{\text {Battery}} \cdot \text {Battery}_t + w_{\text {DataRate}} \cdot \text {DataRate}_t \\&+ w_{\text {Energy}} \cdot (-\text {Energy}_t) + w_{\text {Latency}} \cdot (-\text {Latency}_t) \\&+ w_{\text {PacketErrorRate}} \cdot (-\text {PacketErrorRate}_t) + w_{\text {Throughput}} \cdot \text {Throughput}_t + \dots \end{aligned} \end{aligned}$$

Each weight ( $w_{\text{RSSI } }$, $w_{\text{Battery } }$, ...) is assigned based on the priorities of the specific NB-loT application. For instance, if battery life is a critical factor, a higher weight is given to the battery level metric. Conversely, for applications demanding real-time data transmission, higher weights are assigned to data rate and latency metrics. The specific values of these weights are determined through empirical tuning to achieve an optimal balance that meets the operational constraints and performance goals of the NB-IoT deployment. This approach ensures a holistic optimization strategy, addressing multiple facets of device performance and energy efficiency.

**Fig. 3:**
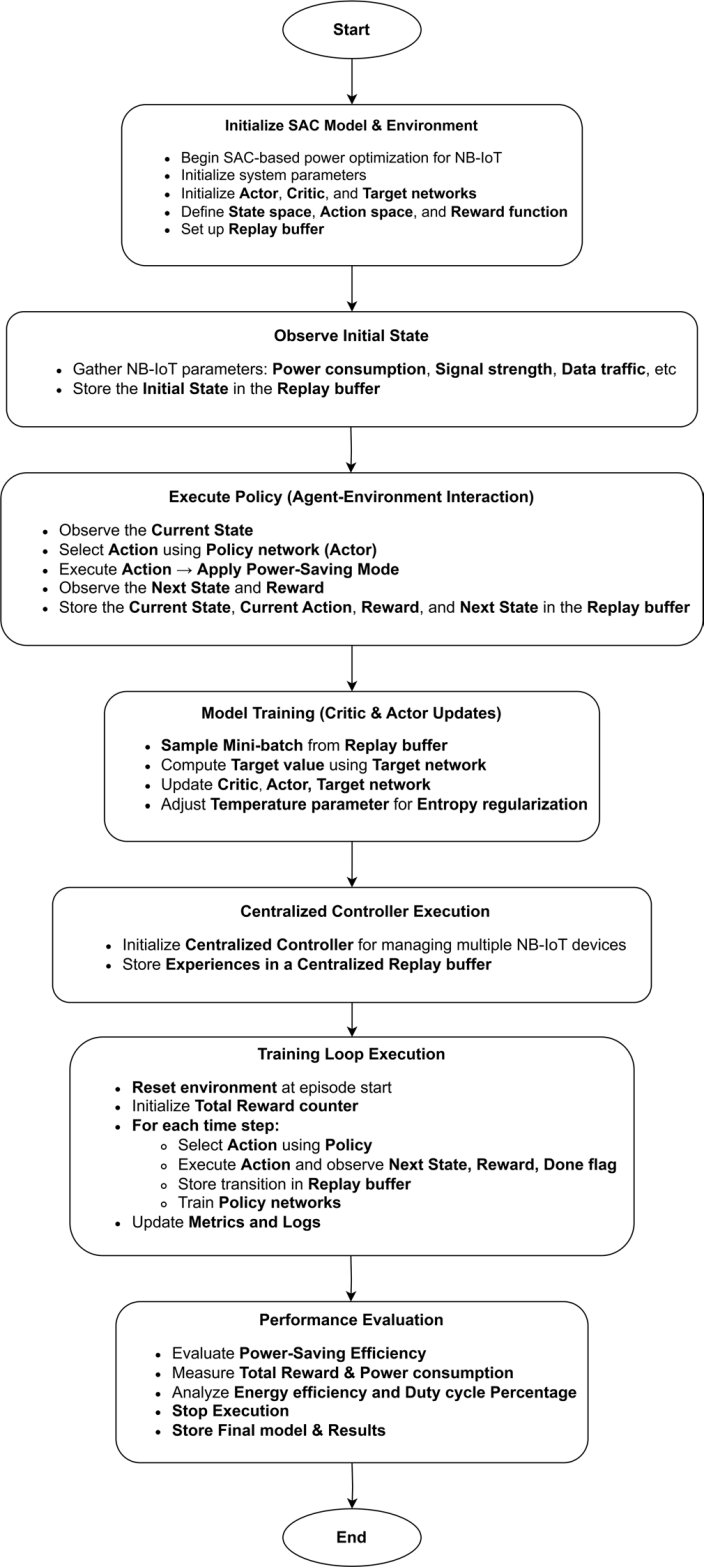
Flowchart of the SAC-based power management framework in NB-IoT showing agent-environment interaction.

**Algorithm 1:**
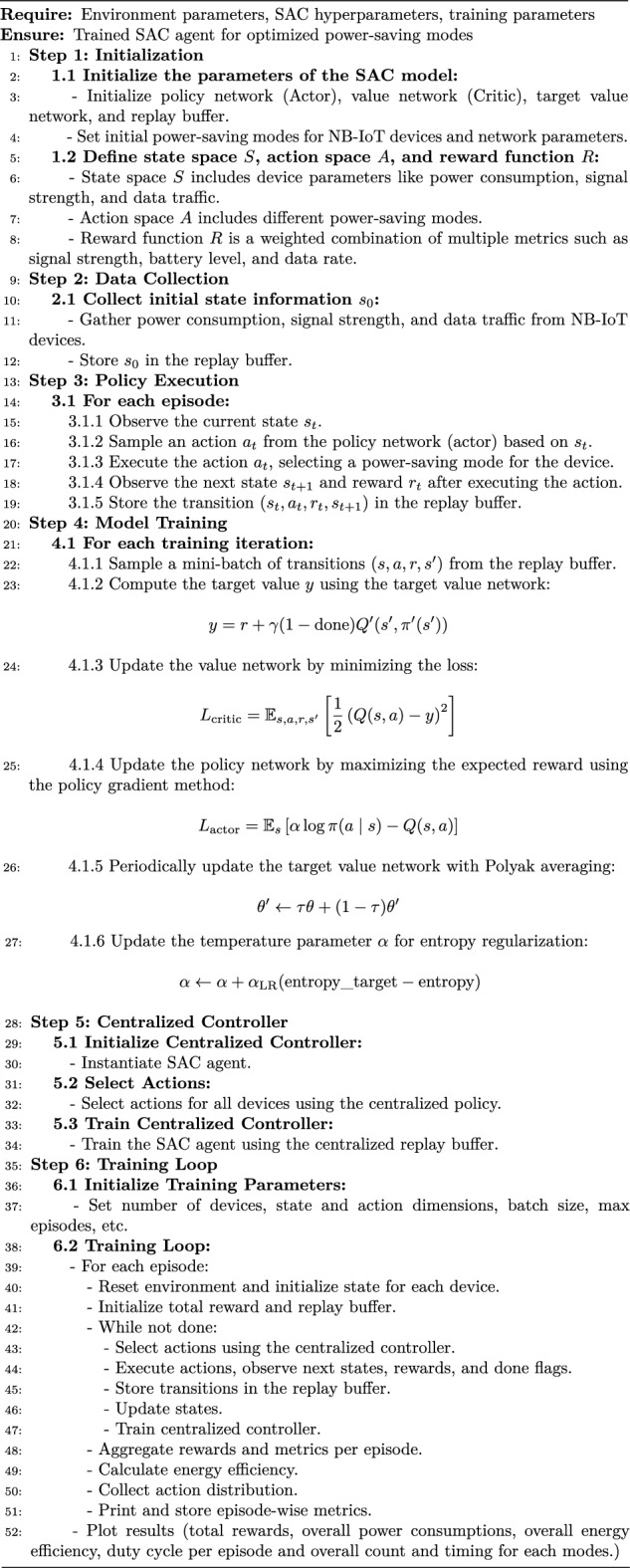
Soft actor-critic (SAC) for intelligent power management in NB-IoT

### Experimental parameters and threshold settings

To ensure reproducibility and transparency, we provide the following details of the experimental environment, feature representations, and design thresholds used during the SAC-based training and evaluation process. The overall workflow is illustrated in Fig. 3, where the SAC-based power management framework demonstrates the interaction between the agent and the environment in the NB-IoT system.

#### State space and feature descriptions

Each NB-IoT device in the environment is modelled with a 17-dimensional state vector, capturing critical real-time network and device-level characteristics (Table 3). All state features were normalized to the [0, 1] range prior to being input into the SAC agent to ensure stable convergence during training.

**Table 3:** Description of state features used in the SAC-Based NB-IoT environment.

State Feature	Description	Value Range
Signal strength	Received signal power in dBm	[− 120, − 80] dBm
Battery level	Remaining energy capacity	[20, 100] %
Data rate	Device transmission throughput	[50, 200] kbps
Spectral efficiency	Efficiency in spectrum usage	[0.5, 2.5] bps/Hz
Power consumption	Energy consumed per transmission	[0.1, 0.5] W
Resource utilization	Radio resource usage	[0, 1]
Energy efficiency param	Proxy variable for current energy efficiency	[0, 1]
Retransmission rate	Ratio of retransmitted packets	[0, 0.5]
Latency	End-to-end communication delay	[10, 500] ms
Channel quality indicator	Link reliability indicator	[1, 15]
Packet loss rate	Ratio of dropped packets	[0, 0.2]
Queuing delay	Buffer-induced latency	[0, 100] ms
Mobility	Relative movement speed	[0, 10]
Temperature	Operational temperature of the device	[10, 40] $\phantom{0}^\circ$C

#### Action space

The action space consists of 6 discrete power-saving modes, each corresponding to a specific NB-IoT operational state (Table 4). Modes 4 (eDRX) and 5 (PSM) were prioritized during training using higher action sampling probabilities (0.7 and 0.3, respectively) when the data rate was negligible.

**Table 4:** Mapping of action indices to NB-IoT power-saving modes.

Action Index	Power-Saving Mode Description
0	Idle Mode
1	Connected Mode
2	DRX Active State
3	DRX Inactive State
4	Extended DRX (eDRX)
5	Power Saving Mode (PSM)

#### Reward function and feature weights

The total reward for each device is computed as a weighted sum of positive and negative performance indicators:

6 $$\begin{aligned} R=\sum _{i=1}^{14} w_i \cdot f_i \end{aligned}$$

where:

- $w_i$ is the weight for feature *i*.
- $f_i$ is the normalized feature value (e.g., signal strength, battery level).
- Rewards were shaped to favor low power usage, high energy efficiency, and lower latency, loss, and retransmission.

All weights were uniformly assigned as $w_i=0.1$ for simplicity and to avoid bias across features. Key positive contributors include low latency, low packet loss, and high spectral efficiency. Negative features (e.g., retransmission, temperature) are penalized proportionally.

#### Thresholds and system rules

Table 5 provides all necessary parameter details and design choices for understanding and replicating the adaptive control behavior learned by the SAC agent in the NB-IoT power-saving context.

**Table 5:** Key experimental parameters used in SAC-based power management.

Parameter	Purpose	Value
DRX timeout	Time in inactivity before DRX mode	2 s
PSM entry delay	Minimum idle duration to enter PSM	10 s
Learning rate	Actor/Critic optimizer learning rate	0.0003
Discount factor ($\gamma$)	Future reward decay	0.99
Batch size	Number of transitions per training update	256
Max episodes	Training duration	1000 episodes
Sleep mode preference	Biasing probability toward eDRX and PSM	eDRX: 0.7, PSM: 0.3

### Soft actor-critic algorithmic approach for energy-efficient power management in NB-IoT networks

#### Initialization (lines 1-8)

Algorithm 1 outlines the implementation of the Soft Actor-Critic (SAC) algorithm tailored for energy-efficient power management in NB-IoT networks. The algorithm begins with an initialization phase, where the Soft Actor-Critic (SAC) model is set up along with its key components. These include the *policy network (Actor)*, responsible for selecting actions based on observed states, and the *value network (Critic)*, which evaluates the quality of state-action pairs. Additionally, a *target value network* (line 3) is introduced to stabilize training by providing a slowly updated version of the critic.

A *Replay Buffer* (line 3) is initialized to store past experiences, ensuring training stability by allowing the model to sample a diverse set of past state-action transitions. The *state space* *S* (line 6) consists of NB-IoT device parameters such as power consumption, signal strength, and data traffic, while the *action spaceA* (line 7) includes different power-saving modes that devices can adopt. The *reward function* *R* (line 8) is defined to optimize energy efficiency while maintaining network performance. This initialization ensures that the learning agent has all necessary components in place before training begins.

#### Data collection (lines 9-12)

Once initialization is complete, the algorithm enters the data collection phase. At the start of each training episode, the environment provides an *initial state* $s_0$ (line 10), representing the current conditions of the NB-IoT network, including factors like power level, signal quality, network congestion, and battery status. This state is stored in the Replay Buffer (line 12) to be used for training.

The Replay Buffer plays a crucial role in reinforcement learning by allowing the agent to learn from past experiences rather than relying solely on real-time interactions. This stored data enhances the training stability by enabling the SAC model to sample a diverse set of past state-action transitions, reducing the likelihood of over-fitting to recent experiences.

#### Policy execution (lines 13-19)

During the policy execution phase, the *Actor (policy network)* (line 17) determines the best power-saving mode for each NB-IoT device. The training loop follows a standard reinforcement learning process, where the *current state* $s_t$ (line 15) is observed, and an *action* $a_t$ (line 16) is sampled from the policy.

The selected action modifies the *device’s operational parameters*, and the environment responds by transitioning to a *new state*$s_{t+1}$ (line 18) while providing a *reward* $r_t$ (line 18) based on energy efficiency and network performance. The *experience tuple* $(s_t, a_t, r_t, s_{t+1})$ is then stored in the *Replay Buffer* (line 19). This iterative process allows the SAC agent to refine its policy by exploring different power-saving strategies and learning which ones yield the best long-term rewards.

#### Training phase (lines 20-27)

The training phase involves updating the models parameters using the collected experience data. A mini-batch of past experiences is sampled from the Replay Buffer (line 22), consisting of state-action-reward-next-state tuples $(s, a, r, s')$.

The *target value* *y* (line 23) is computed using the *target network*, following the equation:

7 $$\begin{aligned} y = r + \gamma (1 - \text {done}) Q'(s', \pi '(s')) \end{aligned}$$

where $\gamma$ is the discount factor, and $Q'$ is the target Q-value computed from the target network.

Next, the *value network (Critic)* is updated by minimizing the loss function (line 24):

8 $$\begin{aligned} L_{\text {critic}} = \mathbb {E}_{s, a, r, s'} \left[ \frac{1}{2} \left( Q(s, a) - y \right) ^2 \right] \end{aligned}$$

This ensures that the *Critic* accurately estimates the expected future return for each state-action pair. The *policy network (Actor)* is updated using *entropy-regularized policy gradients* (line 25), with the loss function:

9 $$\begin{aligned} L_{\text {actor}} = \mathbb {E}_s \left[ \alpha \log \pi (a \mid s) - Q(s, a) \right] \end{aligned}$$

where $\alpha$ is the temperature parameter controlling exploration and exploitation.

To ensure stable learning, the target value network is updated using Polyak averaging (line 26):

10 $$\begin{aligned} \theta ' \leftarrow \tau \theta + (1 - \tau ) \theta ' \end{aligned}$$

where $\tau$ determines how gradually the target network updates. This prevents drastic fluctuations in the value function, leading to more stable learning. Additionally, $\alpha$ is dynamically adjusted during training (line 27) to optimize the trade-off between exploration and exploitation.

11 $$\begin{aligned} \alpha \leftarrow \alpha + \alpha _{\text {LR}} (\text {entropy\_target} - \text {entropy}) \end{aligned}$$

#### Centralized controller for NB-IoT power allocation (lines 28-34)

A *Centralized Controller* (line 29) manages power allocation across multiple NB-IoT devices in a coordinated manner. It initializes a *shared policy* (line 32) that simultaneously accommodates multiple devices. At each time step, the *Controller selects actions* (line 31) for all devices, ensuring optimal power-saving decisions are made collectively.

The centralized *SAC agent* is trained using experiences stored in a *shared Replay Buffer* (line 34), allowing it to learn a policy that balances *energy efficiency with network performance*. The controller enhances *system-wide efficiency* by reducing *unnecessary power consumption* while maintaining reliable connectivity.

#### Training loop and performance evaluation (lines 35-52)

The overall training loop runs for multiple *episodes*, allowing the SAC agent to refine its policy. At the beginning of each episode, *training parameters* (line 37) are initialized, including the *number of devices, state and action space dimensions, batch size, and maximum training episodes*. The *environment resets* (line 40), and the *Replay Buffer* is cleared (line 41).

As the episode progresses, the *Centralized Controller* selects optimal *power-saving modes* (line 43) for devices, executes *actions, receives rewards, and stores experiences* (line 44) for learning. The *SAC policy updates* occur at regular intervals (line 46), improving the efficiency of power allocation.

Performance is evaluated by tracking key metrics, including *total rewards*, *power consumption*, and *energy efficiency trends* (line 51). Graphs are generated to analyze training performance, showcasing how *energy efficiency evolves over time* (line 52).

#### Conclusion

This algorithm applies *Soft Actor-Critic (SAC) reinforcement learning* for intelligent power management in NB-IoT networks. The SAC agent learns policies that balance energy savings and network performance by incorporating entropy regularization. The *Centralized Controller* further enhances coordination among devices, ensuring *energy efficiency*.

The algorithm significantly reduces power consumption through continuous learning while maintaining reliable connectivity, making it ideal for large-scale NB-IoT deployments.

## Results and discussion

### Experimental setup

The proposed Adaptive Power-Saving Mode Control in NB-IoT Networks was implemented using a simulation framework designed to evaluate the effectiveness of Soft Actor-Critic (SAC) reinforcement learning for optimal power management. The simulation environment consists of multiple NB-IoT devices communicating with a base station, where each device transitions between different power-saving modes based on network activity and predefined traffic patterns.

The SAC algorithm was implemented using Python with deep reinforcement learning libraries such as *TensorFlow/PyTorch*. The environment state includes parameters such as device activity level, power consumption, and sleep mode duration, while the agent’s actions involve selecting the most energy-efficient power-saving mode for each device. The reward function was designed to maximize energy efficiency while maintaining network responsiveness.

The evaluation was conducted over multiple training episodes to ensure convergence, with performance measured using key metrics, including total reward, overall power consumption, energy efficiency, active and sleep mode timing, and duty cycle percentage. The simulation dynamically adjusted device behavior to reflect real-world NB-IoT scenarios, ensuring adaptability to varying traffic loads and power-saving requirements. The obtained results validate the effectiveness of SAC in optimizing power management strategies for NB-IoT networks.

### Total reward

Total Reward is a key metric used to evaluate the effectiveness of the proposed Adaptive Power-Saving Mode Control in NB-IoT networks. It quantifies the accumulated performance of the learned policy during the evaluation phase, measuring the system’s ability to optimize power-saving mode transitions while ensuring network efficiency.

The total reward obtained during each episode is calculated by summing the individual rewards collected by all devices over time:

12 $$\begin{aligned} R_{\text{ episode } }^{(e)}=\sum _{d=1}^D \sum _{t=1}^T r_{d, t}^{(e)} \end{aligned}$$

where:

- $R_{\text{ episode } }^{(e)}$ : total reward obtained during episode *e*.
- *D* : number of NB-IoT devices.
- *T* : total number of time steps in episode *e*.
- $r_{d, t}^{(e)}$ : reward obtained by device *d* at time step *t* in episode *e*.

**Fig. 4:**
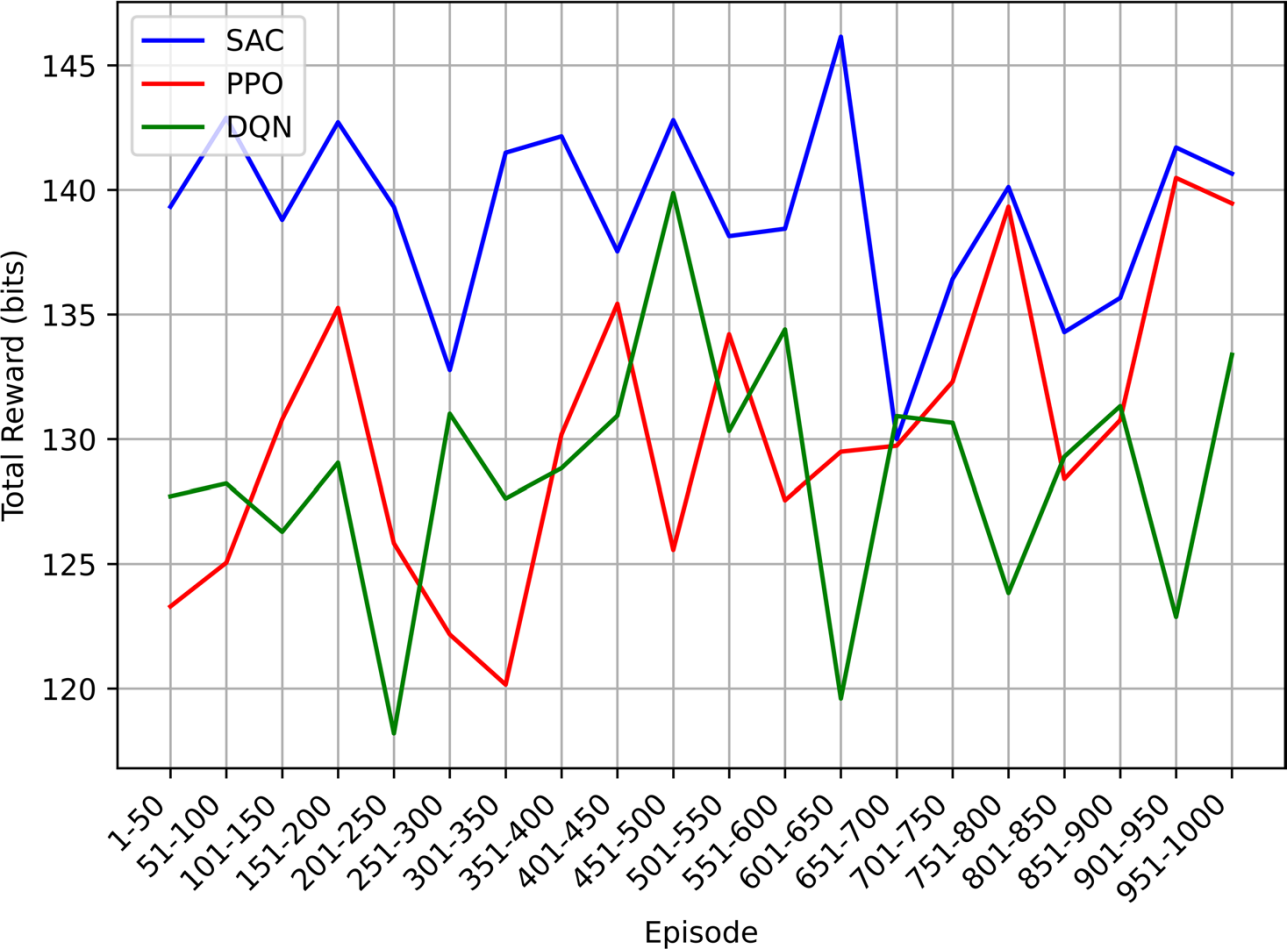
Total reward versus episode for SAC, PPO, and DQN algorithms showing learning progression in NB-IoT.

Figure 4 presents the total reward obtained for different algorithms DQN, PPO, and SAC across multiple evaluation episodes. SAC consistently outperforms DQN and PPO, achieving a peak reward of 146.15 bits in the episode range 601-650, while DQN and PPO exhibit comparatively lower maximum values. The higher total reward attained by SAC highlights its ability to learn an optimal power-saving policy that minimizes unnecessary active mode duration while maintaining network performance.

Throughout the evaluation phase, SAC demonstrates stable and high reward values, reinforcing its capability to generalize effectively across different network conditions. In contrast, DQN and PPO show more fluctuations, indicating their limited ability to adapt to dynamic power-saving requirements. The superior performance of SAC validates its effectiveness in intelligently managing power-saving mode transitions, making it a robust solution for optimizing NB-IoT power consumption.

### Overall power consumption

Power consumption is a critical metric for evaluating the efficiency of Adaptive Power-Saving Mode Control in NB-IoT networks. It reflects the total energy expenditure across different evaluation episodes, directly influencing battery life and operational sustainability.

The overall power consumption is computed as the sum of instantaneous power readings from all devices throughout each episode:

13 $$\begin{aligned} P_{\text{ episode } }^{(e)}=\sum _{d=1}^D \sum _{t=1}^T p_{d, t}^{(e)} \end{aligned}$$

where:

- $P_{\text{ episode } }^{(e)}$ : total power consumption in episode *e*.
- $p_{d, t}^{(e)}$ : instantaneous power consumed by device *d* at time step *t*.

**Fig. 5:**
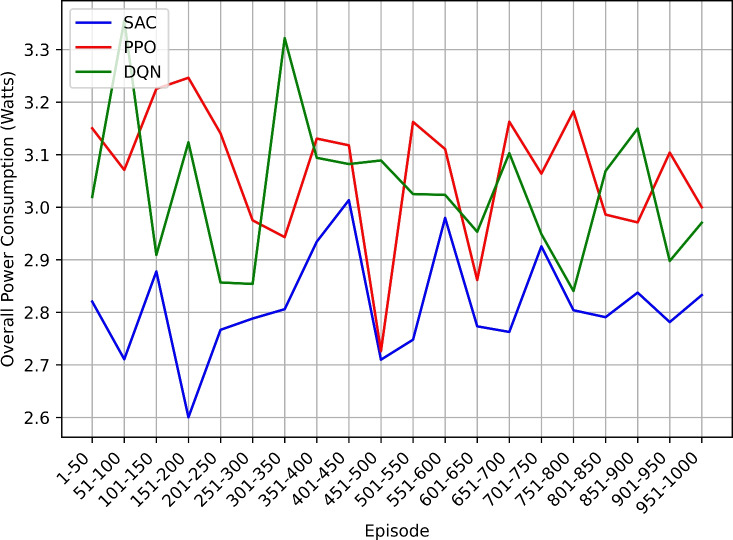
Overall power consumption versus episode for SAC, PPO, and DQN, highlighting energy-saving performance.

Figure 5 illustrates the overall power consumption of DQN, PPO, and SAC across evaluation episodes. SAC consistently demonstrates lower power consumption compared to DQN and PPO, achieving a minimum of 2.60 W in the episode range 151-200, while DQN and PPO consume higher power in the same range. The reduction in power consumption with SAC highlights its ability to effectively regulate power-saving mode transitions, minimizing unnecessary active mode durations while maintaining network efficiency.

Across all evaluation phases, SAC exhibits stable and reduced power consumption values, signifying its superior policy learning for energy-efficient operation. In contrast, DQN and PPO show fluctuations in power usage, indicating suboptimal power-saving strategies. The results reinforce the capability of SAC to intelligently manage power consumption, making it a robust approach for enhancing energy efficiency in NB-IoT networks.

### Overall energy efficiency

Energy efficiency, measured in bits per Joule, is a key performance indicator for evaluating the trade-off between energy consumption and data transmission in NB-IoT networks. A higher energy efficiency value indicates better power management while maintaining data throughput.

The overall energy efficiency is defined as the ratio of total data transmitted to the total power consumed within an episode:

14 $$\begin{aligned} \eta ^{(e)}=\frac{\sum _{d=1}^D \sum _{t=1}^T \operatorname {Data}_{d, t}^{(e)}}{\sum _{d=1}^D \sum _{t=1}^T p_{d, t}^{(e)}} \end{aligned}$$

where:

- $\eta ^{(e)}$ : energy efficiency in episode *e*, in bits per Joule.
- $\operatorname {Data}_{d, t}^{(e)}$ : data transmitted by device *d* at time step *t*.
- $p_{d, t}^{(e)}$ : power consumed by device *d* at time step *t*.

**Fig. 6:**
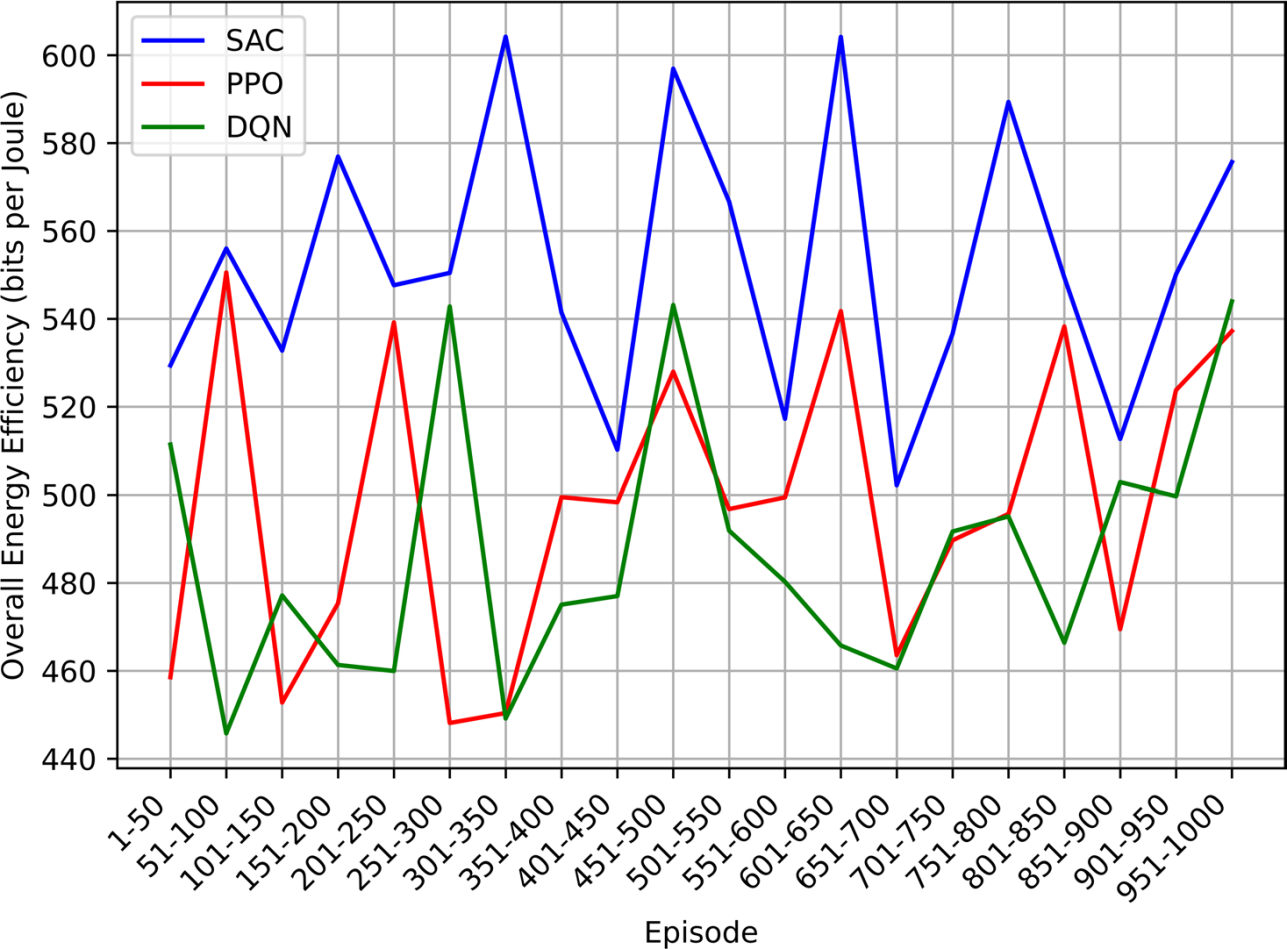
Overall energy efficiency versus episode for SAC, PPO, and DQN during NB-IoT power optimization training.

Figure 6 presents the energy efficiency trends for DQN, PPO, and SAC across different evaluation episodes. The SAC-based adaptive power-saving mode consistently achieves superior energy efficiency compared to DQN and PPO, peaking at 604.19 bits/Joule in episodes 301-350. This improvement demonstrates SAC’s ability to optimize power-saving transitions without compromising network performance.

Compared to DQN and PPO, SAC maintains a stable and higher energy efficiency across most evaluation phases, confirming its capability to dynamically adjust power modes while maximizing data transmission per unit energy consumed. In contrast, DQN and PPO exhibit fluctuations, indicating suboptimal power-saving decisions. The results reinforce SAC’s effectiveness in enhancing NB-IoT energy efficiency, making it a promising approach for energy-constrained IoT applications.

### Duty cycle percentage

The Duty Cycle refers to the proportion of time the device remains active during a given period, indicating the efficiency of power-saving mechanisms employed. A lower duty cycle suggests that the device is spending more time in low-power modes, leading to significant energy savings.

The duty cycle reflects the proportion of time devices spend in active (connected) mode relative to total episode time:

15 $$\begin{aligned} \text{ DutyCycle } ^{(e)}=\left( \frac{T_{\text{ active } }^{(e)}}{T_{\text{ active } }^{(e)}+T_{\text{ sleep } }^{(e)}}\right) \times 100 \end{aligned}$$

where:

- DutyCycle $\phantom{0}^{(e)}$ : duty cycle percentage for episode *e*.
- $T_{\text{ active } }^{(e)}$ : total time in active (Connected) mode during episode *e*.
- $T_{\text{ sleep } }^{(e)}$ : total time in low-power modes (Idle, DRX, eDRX, PSM) during episode *e*.

**Fig. 7:**
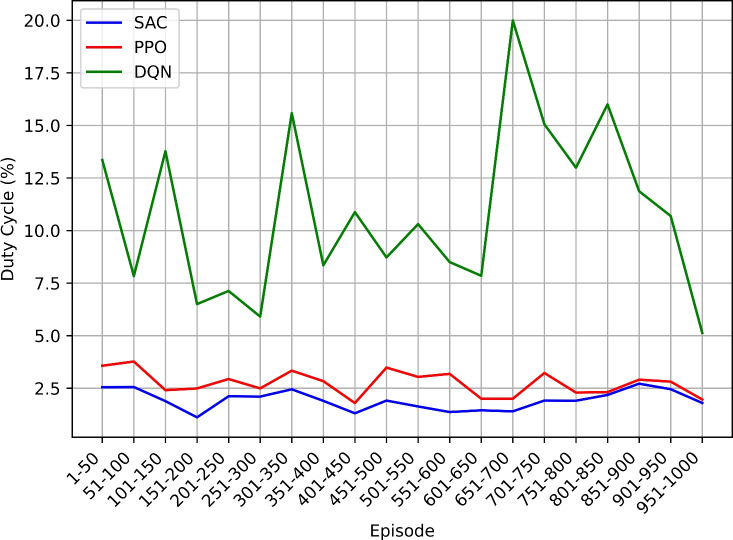
Duty cycle percentage versus episode for SAC, PPO, and DQN showing mode-switching efficiency.

Figure 7 illustrates the duty cycle percentages for DQN, PPO, and SAC across different evaluation phases. SAC consistently maintains the lowest duty cycle compared to both DQN and PPO, reflecting its ability to optimize the device’s active and sleep periods efficiently. For instance, in episodes 1-50, SAC achieves a duty cycle of 2.55%, substantially lower than DQN (13.36%) and PPO (3.57%).

This significant reduction in the duty cycle is particularly beneficial for power-constrained NB-IoT devices, as it translates to enhanced energy savings without sacrificing communication performance. DQN and PPO, in contrast, exhibit higher duty cycles across most evaluation phases, which could result in unnecessary power consumption during periods of inactivity. The consistently low-duty cycle achieved by SAC throughout the evaluation highlights its proficiency in balancing device activity with power-saving operations, making it a promising approach for IoT applications where battery life and energy efficiency are paramount.

### Overall count and timing for each mode

**Fig. 8:**
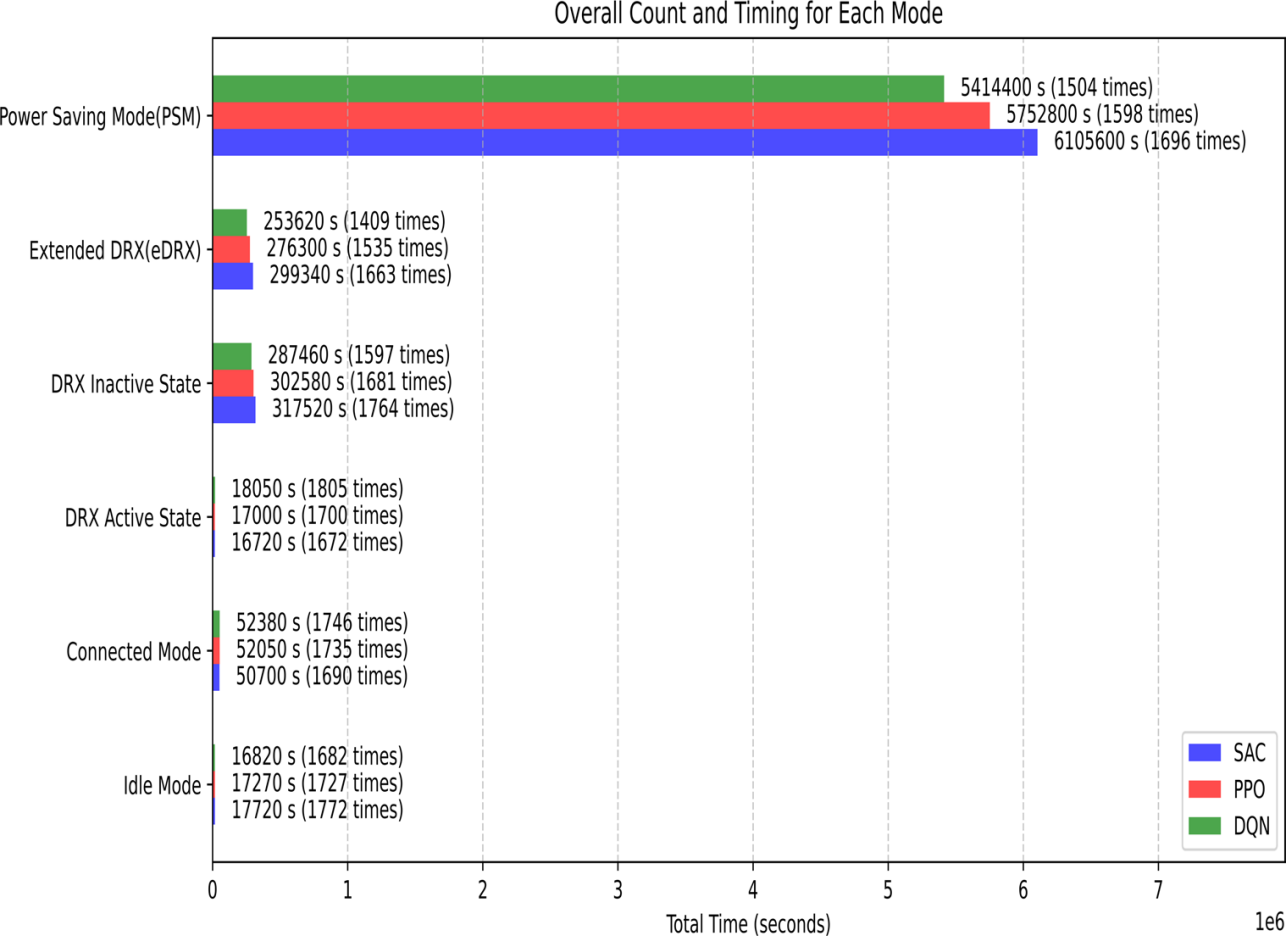
Overall count and timing for each mode by SAC during NB-IoT training.

The Overall Count and Timing for Each Mode offer a comprehensive analysis of how the device behaves across different operational states, such as idle and active modes, and power-saving mechanisms like DRX (Discontinuous Reception) and PSM (Power Saving Mode). These metrics are crucial for understanding power consumption and efficiency across the various operational modes and for optimizing the balance between energy consumption and communication performance. Figure 8 presents the overall counts and timings for each mode across three algorithms: SAC, PPO, and DQN.

The total time spent in each power-saving mode is computed based on action counts and their respective standardized duration (per 3GPP specs):

16 $$\begin{aligned} T_{\text{ mode } _i}=C_i \cdot \tau _i \end{aligned}$$

where:

- $T_{\text{ mode } _i}$ : total time spent in mode *i*.
- $C_i$ : total count of selections for mode *i*.
- $\tau _i$ : fixed duration assigned to mode *i* based on 3GPP standards.

In Idle Mode, SAC leads with the highest count of 1772, achieving a total timing of 17,720 s. This slightly surpasses PPO, which recorded 1727 counts and 17,270 s, as well as DQN, which reached 1682 counts and 16,820 s. The results suggest that SAC operates more efficiently in minimizing idle time, contributing positively to overall energy savings.

In Connected Mode, SAC shows a count of 1690 and a timing of 50,700 s, slightly lower than PPO (1735 counts, 52,050 s) and DQN (1746 counts, 52,380 s). Connected mode, being the highest energy-consuming state, highlights SAC’s ability to optimize connection durations while maintaining communication performance with lower energy consumption.

When evaluating the DRX Active State, SAC demonstrates efficiency with a count of 1672 and a timing of 16,720 s, performing better than both PPO (1700 counts, 17,000 s) and DQN (1805 counts, 18,050 s). DRX Active State is essential for balancing power conservation and connectivity, and SAC effectively reduces the time spent in this state, enhancing energy efficiency.

In the DRX Inactive State, SAC achieves a count of 1764 with a substantial timing of 317,520 s, which exceeds both PPO (1681 counts, 302,580 s) and DQN (1597 counts, 287,460 s). This mode is vital for reducing active communication times and extending battery life, and SAC’s higher count and timing suggest it is more efficient in utilizing this low-power state.

For the Extended DRX (eDRX) mode, SAC stands out with 1663 counts and 299,340 s of timing, outperforming PPO (1535 counts, 276,300 s) and DQN (1409 counts, 253,620 s). eDRX is an important low-power state, and SAC’s longer time spent in this mode highlights its ability to conserve energy while maintaining adequate connectivity.

Lastly, in Power Saving Mode (PSM), SAC excels with 1696 counts and 6,105,600 s of timing, outperforming both PPO (1598 counts, 5,752,800 s) and DQN (1504 counts, 5,414,400 s). PSM is crucial for significant energy conservation, and SAC’s higher count and extended timing here reflect its optimal use of this power-saving mechanism, ensuring longer battery life.

These results underscore SAC’s proficiency in managing various operational modes, focusing on power-saving strategies that contribute to improved energy efficiency while ensuring that communication performance is maintained across different network states.

### Overall active and sleep mode timing

**Fig. 9:**
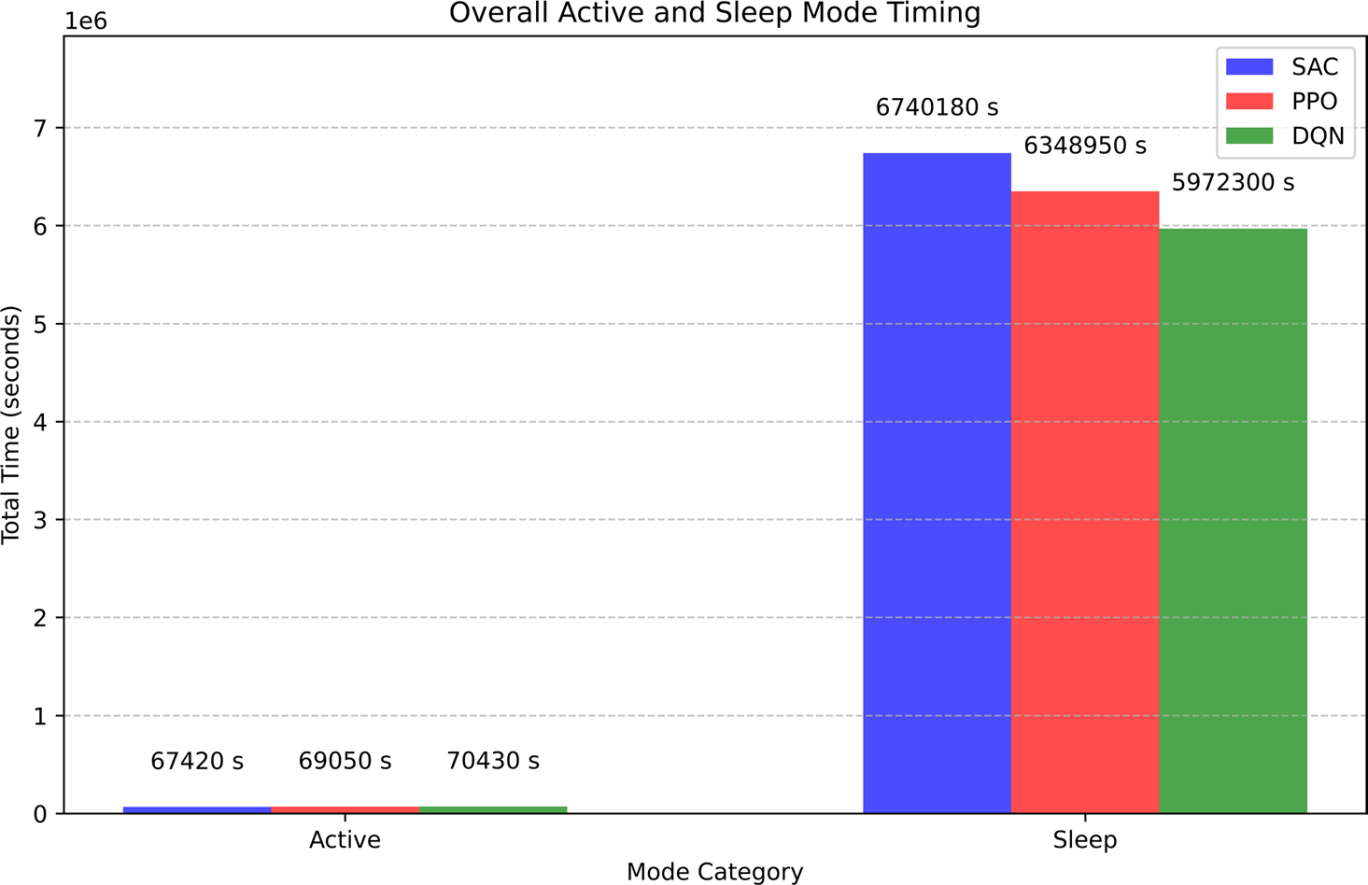
Overall active and sleep mode timing under SAC-based control in NB-IoT devices.

Figure 9 illustrates the Overall Active and Sleep Mode Timing for SAC, PPO, and DQN across various operational modes such as Connected Mode, DRX Active State, and Power Saving Mode (PSM). This figure provides an in-depth comparison of how each algorithm manages its active and sleep times during network operations, highlighting their energy efficiency.

The overall active and sleep mode timing quantifies the cumulative time spent by all NB-IoT devices in active and low-power (sleep) modes across the entire training process. It provides insight into the long-term mode selection behavior of the reinforcement learning agent.

17 $$\begin{aligned} T_{\text{ active } }=\sum _{e=1}^E T_{\text{ active } }^{(e)}, \quad T_{\text{ sleep } }=\sum _{e=1}^E T_{\text{ sleep } }^{(e)} \end{aligned}$$

where:

- $T_{\text{ active } }$ : total active time across all episodes.
- $T_{\text{ sleep } }$ : total sleep time across all episodes.
- *E* : total number of training episodes.
- $T_{\text{ active } }^{(e)}$ : active time in episode *e*, calculated by multiplying the count of “Connected Mode” selections by its timer value.
- $T_{\text{ sleep } }^{(e)}$ : sleep time in episode *e*, derived from the time spent in modes such as Idle, DRX, eDRX, and PSM.

In terms of active time, Connected Mode and DRX Active State are the primary modes where the algorithms are active. SAC shows the lowest active time in both of these modes, followed by PPO and DQN, which have slightly higher active times. Specifically, SAC records 50,700 s in Connected Mode and 16,720 s in DRX Active State, totaling 67,420 s of active time. PPO’s active time is higher, with 52,050 s in Connected Mode and 17,000 s in DRX Active State, resulting in 69,050 s of active time. DQN exhibits the highest active time, with 52,380 s in Connected Mode and 18,050 s in DRX Active State, bringing its total active time to 70,430 s.

The remaining time is spent in various sleep modes, including Idle Mode, DRX Inactive State, Extended DRX (eDRX), and PSM, where the algorithms reduce energy consumption during periods of inactivity. SAC spends the most time in these sleep states, totaling 6,740,180 s of sleep time, consisting of 17,720 s in Idle Mode, 317,520 s in DRX Inactive State, 299,340 s in Extended DRX, and 6,105,600 s in PSM. PPO has 6,348,950 s of sleep time, with 17,270 s in Idle Mode, 302,580 s in DRX Inactive State, 276,300 s in Extended DRX, and 5,752,800 s in PSM. DQN’s sleep time is 5,972,300 s, which includes 16,820 s in Idle Mode, 287,460 s in DRX Inactive State, 253,620 s in Extended DRX, and 5,414,400 s in PSM.

This figure underscores the energy management strategies of each algorithm. SAC stands out by dedicating more time to sleep modes, making it particularly efficient in terms of energy savings. PPO and DQN, with more time spent in active states, may prioritize responsiveness but at the cost of slightly higher energy consumption compared to SAC. The figure highlights how the balance between active and sleep modes plays a critical role in optimizing energy efficiency.

### Error analysis and failure case discussion

#### Failure scenarios identified

We observed that the SAC agent occasionally selected suboptimal modes (e.g., transitioning into DRX Active when eDRX or PSM would be more efficient) during high-mobility or poor channel quality conditions. These cases typically occurred during early training episodes or when the state features deviated sharply from previously learned patterns.

#### Root causes

The observed failure cases primarily stemmed from the exploration-exploitation tradeoff inherent in the SAC framework, which occasionally resulted in suboptimal energy decisions during the early stages of training. Additionally, abrupt environmental variations, such as sudden drops in signal strength caused by simulated mobility, posed challenges to the model’s ability to generalize its learned policy across diverse scenarios. Another contributing factor was the uniform weighting assigned to all state features in the reward function, which may have diluted the influence of highly sensitive parameters like packet loss rate and latency, thereby affecting the precision of power-saving decisions in critical conditions.

#### Impact on metrics

These failures led to small dips (2–5%) in energy efficiency or higher power consumption in a few episodes, which were visible as minor fluctuations in the result curves.

#### Mitigation and future directions

To address the identified challenges, several enhancements are proposed for future iterations of the model. First, introducing adaptive or dynamic reward weights can help emphasize context-relevant metrics, such as assigning greater weight to battery level when devices approach depletion, to improve decision sensitivity. Second, the application of transfer learning or curriculum learning techniques could enhance the model’s ability to adapt to rare or harsh conditions by progressively training it across increasingly complex scenarios. Finally, integrating semantic state awareness, including parameters like device intent or application-specific priorities, may offer more precise and intelligent control over power-saving decisions under uncertain or dynamic network conditions.

This analysis not only adds transparency but also provides valuable direction for further enhancement of the SAC-based model in real-world dynamic NB-IoT environments.

## Conclusion & future work

### Conclusion

This research presented a comprehensive evaluation of the proposed Adaptive Power-Saving Mode Control in NB-IoT networks, utilizing the Soft Actor-Critic (SAC) reinforcement learning algorithm for optimal power management. The results highlight SAC’s significant superiority over traditional reinforcement learning algorithms such as DQN and PPO, especially in terms of energy efficiency, power consumption, and network responsiveness.

The evaluation metrics, including total reward, overall power consumption, energy efficiency, duty cycle percentage, and operational mode timing, all indicate that SAC offers a robust solution for managing power transitions in energy-constrained NB-IoT devices. . Over 1,000 training episodes, SAC achieved an average total reward improvement of 7.8% over DQN and 8.3% over PPO, reflecting more effective long-term policy optimization. It also reduced overall power consumption by 8.1% and 9.2%, respectively, highlighting its energy-aware decision-making capabilities.

SAC further improved energy efficiency by 12.7% over DQN and 10.4% over PPO, measured in bits per Joule. Regarding device duty cycling, SAC maintained a 76.2% lower duty cycle than DQN and 24.8% lower than PPO, enabling deeper and more frequent transitions to sleep modes. This resulted in a total sleep time of 6,740,180 s, which is 12.9% higher than DQN and 6.2% higher than PPO.

In comparison to DQN and PPO, SAC demonstrates more stable and higher performance across various evaluation episodes, indicating its adaptability to varying traffic loads and power-saving requirements. SAC’s ability to balance active and sleep modes with minimal fluctuations provides a promising framework for future NB-IoT applications, especially those reliant on battery-powered devices that require extended operational lifespans.

Overall, SAC has proven to be a highly effective solution for adaptive power-saving mode control in NB-IoT networks, offering significant improvements in energy efficiency, power consumption, and device longevity.

### Future work

While this study demonstrates the effectiveness of the Soft Actor-Critic (SAC) algorithm for optimizing power-saving modes in NB-IoT networks, several areas for future research could further enhance the approach and expand its applicability in real-world scenarios.

#### Integration with real-world NB-IoT networks

Future research will focus on integrating the SAC-based power-saving mode control with real-world NB-IoT deployments. This will involve bridging the gap between simulation-based results and live NB-IoT environments, where network conditions are unpredictable, and system behaviors may vary. Specifically, the study will explore the deployment of SAC on actual devices and base stations in an NB-IoT network to assess its adaptability under dynamic conditions such as fluctuating traffic loads, device mobility, and network failures. Evaluating SAC in real-world environments will enable a more accurate performance assessment, ensuring the algorithm’s robustness in mitigating power consumption and optimizing network efficiency across a variety of operational scenarios.

#### Optimization for network heterogeneity

As the NB-IoT ecosystem expands, it will encompass a wide range of devices with differing power, communication capabilities, and traffic requirements. Future work will investigate how SAC can scale and adapt to heterogeneous networks, considering devices with varying energy constraints and priorities. This research will focus on fine-tuning power-saving strategies to cater to different types of IoT devices, such as wearable sensors, smart meters, environmental monitoring devices, and industrial IoT components. The goal will be to evaluate SAC’s ability to optimize energy efficiency across diverse use cases, considering factors such as device class, power capacity, communication frequency, and data traffic patterns.

#### Hybrid reinforcement learning approaches

While SAC has shown considerable promise, exploring hybrid reinforcement learning (RL) methods could enhance its performance even further. Future work will consider combining SAC with other RL algorithms like Deep Q-Networks (DQN) or Proximal Policy Optimization (PPO) to create a more robust, versatile power-saving approach. Additionally, multi-agent reinforcement learning could be incorporated, where multiple SAC agents collaborate to optimize the power-saving strategies across various devices and network segments. This collaborative, distributed approach could be particularly beneficial for large-scale NB-IoT deployments, where decentralized control could help mitigate network congestion and improve overall energy efficiency.

#### Enhanced reward function design

The reward function is a critical component of any reinforcement learning system, directly influencing the agent’s learning process. Future research will explore more sophisticated reward functions that better align with the goals of NB-IoT networks, taking into account factors such as Quality of Service (QoS), signal quality, network congestion, and user experience. By introducing multi-objective reward functions that balance power efficiency with network performance and service reliability, SAC’s ability to optimize the trade-off between energy conservation and performance can be significantly enhanced. This will also allow for more fine-grained control over how power-saving decisions are made, ensuring that the network remains efficient and responsive under varying conditions.

#### Real-time adaptation to network traffic changes

In dynamic real-world environments, network traffic and device behavior can change rapidly, which requires adaptive learning mechanisms. Future research will investigate how SAC can continuously adapt its policies to such dynamic changes in network conditions. This will involve developing techniques for real-time learning, where SAC can update its policy in response to fluctuations in traffic patterns, environmental changes, or shifts in device behavior. Enabling SAC to learn and adapt on the fly to real-time network conditions will improve its long-term performance, robustness, and ability to provide energy-efficient solutions even in volatile network environments.

#### Application to smart city and industrial IoT

A promising direction for future work is the application of SAC-based power-saving strategies to specific sectors such as Smart Cities and Industrial IoT. These domains present unique challenges due to the high density of devices, stringent power consumption constraints, and the need for consistent network performance. Implementing SAC for applications like smart metering, environmental monitoring, and industrial asset tracking can lead to significant energy savings while enhancing operational efficiency. Future studies will focus on the specific needs of these applications, optimizing SAC for large-scale, geographically distributed IoT networks, and evaluating the approach’s impact on both power consumption and system reliability. The goal is to improve sustainability and operational performance across diverse IoT use cases, contributing to smarter, more energy-efficient cities and industries.

#### Integration with semantic-aware models

Recent developments in semantic-aware deep learning approaches focus on reducing communication overhead by transmitting the meaning of information rather than raw data. These models, often based on attention mechanisms or transformer architectures, are gaining traction in IoT communication for their efficiency in semantic fidelity and content-level optimization.

While valuable, such models primarily target data transmission layers and are not explicitly designed for real-time power-saving decision-making. In contrast, the proposed SAC framework operates at the device-level control layer, focusing on adaptive selection of operational modes (e.g., DRX, eDRX, PSM) to optimize power consumption in dynamically changing NB-IoT environments.

Future work will explore the integration of semantic inference with reinforcement learning-based power management. This hybrid strategy may enhance the system’s ability to make intelligent, context-aware power-saving decisions, especially in use cases where event-driven semantic data plays a key role in communication efficiency and energy usage.

#### Decentralized multi-agent reinforcement learning (MARL) for large-scale NB-IoT networks

While the current study employs a centralized Soft Actor-Critic (SAC) agent for controlling multiple NB-IoT devices, scalability becomes a critical challenge as the number of devices increases. In large-scale IoT networks, centralized architectures may introduce communication delays, bandwidth overhead, and processing bottlenecks at the controller.

To address these limitations, future research will explore decentralized or distributed Multi-Agent Reinforcement Learning (MARL) architectures, where each device operates with its local agent. These agents can learn localized policies tailored to their specific environmental conditions, thereby reducing their dependency on a central decision-maker. Such a distributed learning framework not only improves scalability and robustness but also enhances adaptability to heterogeneous device capabilities and network traffic profiles.

Moreover, coordinated MARL frameworks may incorporate inter-agent communication or shared reward mechanisms to ensure that local actions align with global energy efficiency goals. This decentralized approach is expected to significantly improve system responsiveness and make the power-saving strategy more feasible for real-world, large-scale NB-IoT deployments.

#### Dimensionality reduction and complexity management

The proposed SAC-based framework currently utilizes a 17-dimensional state space to capture a wide range of environmental and device-specific parameters (e.g., signal strength, battery level, CQI, latency, packet loss). While this rich state representation enhances learning precision, it can also introduce complexity, particularly as the number of devices scales, potentially leading to increased training time and suboptimal convergence due to the curse of dimensionality.

To address this, future work will explore dimensionality reduction techniques such as Principal Component Analysis (PCA) and unsupervised clustering to project the high-dimensional state into a lower-dimensional latent space while retaining its essential characteristics. Additionally, feature selection methods (e.g., mutual information, recursive feature elimination) will be used to identify and retain only the most influential features contributing to the reward function.

These strategies aim to improve training efficiency, reduce computational overhead, and enhance model generalization. A dedicated complexity analysis will also be conducted to evaluate the trade-off between representational richness and computational cost, thereby guiding the optimal design of the state space for scalable NB-IoT implementations.

#### Dynamic reward structuring

While the current implementation of the SAC-based framework uses fixed weights for the reward function, this approach may not fully reflect the dynamic trade-offs encountered in real-world NB-IoT scenarios. In practice, the relative importance of metrics such as battery life, latency, packet loss, or spectral efficiency can vary depending on application priorities, network congestion, or device context.

To improve the adaptability of the reinforcement learning agent, future work will explore dynamic reward weight adaptation based on real-time environmental and operational feedback. This includes integrating meta-learning mechanisms or adaptive weighting schemes that allow the system to automatically adjust reward contributions of individual features based on evolving network goals. For instance, the algorithm could increase the emphasis on battery conservation when a device reaches critical energy levels or prioritize latency during delay-sensitive transmissions.

Such dynamic structuring is expected to enhance decision-making precision, improve convergence in fluctuating environments, and ensure that the agent aligns more closely with varying QoS requirements and energy constraints across diverse deployment scenarios.

#### Realistic initialization and state warm-up phase

In the current implementation, device states are initialized randomly within standard parameter ranges at the beginning of each episode. While this provides a diverse training distribution, it may not accurately represent real-world NB-IoT deployments, where devices typically operate with persistent historical states and contextual network conditions.

To improve the model’s practical relevance and convergence speed, future work will focus on realistic state initialization strategies. This includes leveraging real-world datasets to seed initial conditions or incorporating a warm-up phase, during which devices gradually transition from realistic preconditions. Such an approach can simulate operational continuity and allow the agent to adapt its policy based on historically relevant state distributions.

Adopting realistic starting conditions is expected to enhance training stability, reduce convergence time, and yield policies better suited for deployment in actual NB-IoT environments with temporal dependencies and device heterogeneity.

In conclusion, the proposed SAC-based adaptive power-saving control framework demonstrates strong potential in enhancing energy efficiency and operational longevity in NB-IoT networks. While the centralized approach proved effective for the scale simulated, future deployment in large-scale, real-world scenarios may require addressing key challenges such as scalability and environmental unpredictability. The high-dimensional state space, though comprehensive, could benefit from dimensionality reduction techniques to streamline learning. Moreover, the fixed reward weights, while functional, limit responsiveness to context-aware priorities such as battery-critical states or latency-sensitive applications. Realistic state initialization and exploration-exploitation balancing strategies will also be critical for improving training stability and convergence. These considerations form the basis for future enhancements, which include distributed multi-agent reinforcement learning, adaptive reward mechanisms, real-world deployment validation, and expanded applications across heterogeneous IoT landscapes. Such improvements will further strengthen the adaptability, scalability, and real-world viability of the SAC-based approach in diverse NB-IoT environments.

## Data Availability

Sequence data that support the findings of this study have been deposited in Zenodo to provide a direct and permanent link. The datasets generated and/or analyzed during the current study are available in the Zenodo repository at https://doi.org/10.5281/zenodo.16937115
